# Whole-Genome-Sequencing characterization of bloodstream infection-causing hypervirulent *Klebsiella pneumoniae* of capsular serotype K2 and ST374

**DOI:** 10.1080/21505594.2017.1421894

**Published:** 2018-02-27

**Authors:** Xiaoli Wang, Yingzhou Xie, Gang Li, Jialin Liu, Xiaobin Li, Lijun Tian, Jingyong Sun, Hong-Yu Ou, Hongping Qu

**Affiliations:** a Department of Critical Care Medicine, Ruijin Hospital, Shanghai Jiao Tong University School of Medicine, Shanghai, China; b State Key Laboratory of Microbial Metabolism, Joint International Laboratory of Metabolic & Developmental Sciences, School of Life Sciences & Biotechnology, Shanghai Jiao Tong University, Shanghai, China; c Department of Laboratory Medicine, Jinshan Hospital, Shanghai Medical College, Fudan University, Shanghai, China; d Department of Laboratory Medicine, Huashan Hospital, Shanghai Medical College, Fudan University, Shanghai, China; e Department of Clinical Microbiology, Ruijin Hospital, Shanghai Jiaotong University School of Medicine, Shanghai, China

**Keywords:** bloodstream infection, capsular serotype K2, comparative genomic analysis, Klebsiella pneumoniae, hypervirulent, ST374

## Abstract

Hypervirulent *K. pneumoniae* variants (hvKP) have been increasingly reported worldwide, causing metastasis of severe infections such as liver abscesses and bacteremia. The capsular serotype K2 hvKP strains show diverse multi-locus sequence types (MLSTs), but with limited genetics and virulence information. In this study, we report a hypermucoviscous *K. pneumoniae* strain, RJF293, isolated from a human bloodstream sample in a Chinese hospital. It caused a metastatic infection and fatal septic shock in a critical patient. The microbiological features and genetic background were investigated with multiple approaches. The Strain RJF293 was determined to be multilocis sequence type (ST) 374 and serotype K2, displayed a median lethal dose (LD50) of 1.5 × 10^2^ CFU in BALB/c mice and was as virulent as the ST23 K1 serotype hvKP strain NTUH-K2044 in a mouse lethality assay. Whole genome sequencing revealed that the RJF293 genome codes for 32 putative virulence factors and exhibits a unique presence/absence pattern in comparison to the other 105 completely sequenced *K. pneumoniae* genomes. Whole genome SNP-based phylogenetic analysis revealed that strain RJF293 formed a single clade, distant from those containing either ST66 or ST86 hvKP. Compared to the other sequenced hvKP chromosomes, RJF293 contains several strain-variable regions, including one prophage, one ICE*Kp*1 family integrative and conjugative element and six large genomic islands. The sequencing of the first complete genome of an ST374 K2 hvKP clinical strain should reinforce our understanding of the epidemiology and virulence mechanisms of this bloodstream infection-causing hvKP with clinical significance.

## Introduction

*Klebsiella pneumoniae* is a major opportunistic pathogen, primarily affecting severe and immunocompromised patients in intensive care units (ICUs), and causing various types of community and hospital-acquired infections [1]. Hospital outbreaks of *K. pneumoniae* infection are often associated with multidrug resistance in the causative strains, in particular for sequence type 11 (ST11) and ST258 isolates determined by multi-locus sequence typing (MLST) [2]. However, during the past three decades, hypervirulent variants of *Klebsiella pneumoniae* (hvKP) have been increasingly reported in the Asian Pacific Rim and Western countries, which cause severe infec-tions such as liver abscess, endophthalmitis, meningitis, osteomyelitis, and necrotizing fasciitis in healthy and ambulatory individuals [3]. Recently, an epidemiological study showed that hvKP was identified in 31% of patients with *K. pneumoniae* bacteremia in Beijing Chao-Yang Hospital, China [4].

In general, a positive string test (with the formation of a 5 mm mucoid string or greater) is employed to quickly examine ropy exopolysaccharide produced by *K. pneumoniae*, which is defined as hypermucoviscosity and used to facilitate distinction of hvKP from the classic *K. pneumoniae* (cKP) [3]. However, new evidence has suggested that hypervirulence and hypermucoviscosity are two complementary but distinct phenotypes of *K. pneumoniae* [5]. Notably, *K. pneumoniae* isolates negative by string test are also capable of producing an invasive infection. The string test is thus insufficient in determining the hypervirulence of a *K. pneumoniae* strain, and more hypervirulence-associated genes have to be explored [5].

Isolates with serotypes K1 and K2 have been found to be the most common in hvKPs. Over 80% of hvKPs causing invasive pyogenic liver abscesses in Taiwan, South Korea, and mainland China were characterized to be of these two serotypes [1,6]. Serotype K1 has been determined to be closely associated with ST23 by MLST. In contrast, the *K. pneumoniae* strains with serotype K2 exhibit diverse MLST types (including ST65, ST66, ST86, ST374, ST375, ST380 and etc.), among which ST65 and ST86 are predominant and with higher virulence than other MLST types [6,7]. It thus requires more genetic parsing to get a deep understanding of the virulence of K2 *K. pneumoniae* compared to K1. To our knowledge, no complete genome sequences for ST374 and serotype K2 isolates are publicly available. Herein, we carried out a retrospective analysis of hvKP isolates collected over 19 months at a hospital setting in China. A hypermucoviscous clinical strain, RJF293, causing bacteremia metastatic infection was characterized as the sequence type ST374 and capsular serotype K2. The microbiological features and genome information (including the presence/absence pattern of virulence genes, mobile genetic elements, etc.) of this strain were characterized with multiple approaches, making up a supplement to the existing knowledge on K2 hvKP.

## Materials and methods

### Ethics and consent

This study was observational, using human bacteria samples for the *ex vivo* experiments and sampling patients' blood as part of routine work in hospital treatment, therefore verbal informed consent was obtained from all volunteers. The bacteria samples and data sheets were anonymized. This study protocol including the verbally informed consent procedure was approved by the ethics committee of the Ruijin Hospital affiliated with Shanghai Jiao Tong University, Shanghai, China (reference number: 201329). Protocols of mouse experiments have been approved by the same ethics committee.

### Bacterial strains, plasmids, and growth media

During the study period of September 2014 to March 2016, *K. pneumoniae* isolates were collected from patients' samples at the Ruijin Hospital, Shanghai, China. *K. pneumoniae* identification was based on biochemical profiling by the VITEK2 compact system and 16S rRNA gene sequencing. The strains were cultivated in LB broth or on LB agar supplemented with the appropriate antibiotics. The detailed information of strains used in this study is listed in Supplementary Table S1.

### String test for the hypermucoviscosity phenotype

The string test to determine the hypermucoviscosity phenotype was performed by touching a colony grown overnight on a blood agar plate at 37°C with a loop and pulling up. Strains exhibiting a mucoid string with length of 5 mm or longer were defined as hypermucoviscous [3].

### PFGE, MLST, K-typing and detection of known virulence genes

The Pulsed-field Gel Electrophoresis (PFGE) protocol used was based on the PulseNet 1-day standardized PFGE protocol for *Escherichia coli* O157:H7, *Salmonella*, and *Shigella* [8]. PFGE patterns were interpreted by using the criteria proposed by Tenover* et al* [9]. A *Salmonella* serotype Braenderup strain (H9812) was used to normalize migration variation occurring across the gel and to determine sample band sizes accurately [10].

MLST was also performed by amplifying and sequencing seven *K. pneumoniae* housekeeping genes, including *gapA, infB, mdh, pgi, phoE, rpoB,* and *tonB,* according to the protocol provided on the Pasteur MLST website [11]. Serotype-specific genes for the K1 and K2 capsular serotypes were also amplified by PCR amplification of the *cps* gene cluster at the *wzy* and *wzx* loci as described previously [12]. The presence of ten known *K. pneumoniae* virulence genes*,* including *magA, rmpA*, *allS*, *mrkD*, *kfuBC*, *cf29a*, *fimH*, *uge*, *wab* and *ureA*, was assessed by PCR [13]. The PCR primers used are listed in Supplementary Table S2.

### Antimicrobial susceptibility testing

The VITEK2 compact system (bioMérieux, Marcy l'Etoile, France) was used for antimicrobial susceptibility testing. Results were interpreted according to the interpretive standards of the Clinical and Laboratory Standards Institute [14]. Carbapenem resistance was defined as resistance to imipenem using an MIC breakpoint of ≥ 4 μg/ml or ertapenem using an MIC breakpoint of ≥ 2 μg/ml following the revision of MIC breakpoints in 2014 [15].

### Mouse lethality assay

Evaluation of *K. pneumonia*e RJF293 was performed with the BALB/c mouse, as described previously [16]. Five-week-old female BALB/c mice were used for the virulence assessment in the present study. Briefly, the overnight culture of the *K. pneumoniae* strains to be tested was inoculated into fresh LB broth at 1:100 and grown for 3 hours in a 37°C shaking incubator. Then the cells were harvested, washed once and resuspended in saline (0.9%) to OD600 of 1.0 (approximately 10^9^ CFU/ml). Serial dilution was done to get the appropriate concentration, which was 10^2^ CFU/ml, 10^3^ CFU/ml, 10^4^ CFU/ml and 10^5^ CFU/ml for the determination of median lethal dosage (LD50), and 10^4^ CFU /ml and 10^5^ CFU/ml for the survival assay. One hundred microliters of the bacterial suspension was injected into the mice intraperitoneally. All mice were monitored daily for survival. For the survival analysis, 10 mice were involved in each group, with 5 per group used for the LD50 determination. The ST11 cKP strain HS11286 and the ST23 hvKP strain NTUH-K2044 were used for controls [17,18]. Kaplan-Meier survival estimation was performed with the R package ‘survival’.

### DNA preparation, genome sequencing and annotation

The RJF293 isolate was grown overnight at 37°C to stationary phase in LB medium, and total DNA was isolated from harvested cells. The genomic DNA was extracted by using the CTAB/phenol-chloroform method that has been described previously with slight modification [19]. The overnight culture of *K. pneumoniae* RJF293 was inoculated into 50 ml fresh LB broth at a ratio of 1:100. Bacteria were harvested by centrifuging when the OD600 of the subculture reached 0.8. The pellet was resuspended in 4 ml TE buffer by both vortexing and pipetting. The suspension was supplemented with 300 μl lysozyme (50 mg/ml) and placed into a 37°C water bath for 1 hour. Then SDS and proteinase K were added to a final concentration of 1% (m/v) and 0.5 mg/ml, respectively. The mixture was incubated at 65°C until completely lysed. One milliliter of NaCl solution (5 mol/L) and 1 ml CTAB/NaCl were added in order. The mixture was incubated at 65°C for 15 min, and then 7 ml phenol: chloroform was added (1:1). The tube was placed on a revolver for at least 1 hour. The supernatant containing DNA was separated by spinning. The application of phenol: chloroform was repeated once to eliminate protein as much as possible. Then, the DNA was precipitated with 0.6 volumes isopropanol and washed with 70% ethanol twice. The quality of the purified genomic DNA was assessed by both electrophoresis and NanoDrop™ 2000 fluorospectrometer (Thermo Fisher Scientific, MA, USA). The sequencing library was prepared using the Illumina Nextera XT DNA Library Prep kit as per the user instructions.

The genome sequence was first determined by using IlluminaMiseq short read sequencing (2 × 251 paired-end sequencing for a 400-bp library) and assembled with the Newbler Assembler, resulting in sequences with 124-fold coverage and 99 assembled contigs. Then, with the PacBio RSII sequencing platform, a 4-kb template was prepared, and the library was sequenced on one SMRT cell, yielding 96,326 direct reads with an average length of ∼4.7 kb. These long reads were assembled by using the PacBio HGAP3 workflow. Finally, the assembled contigs derived from the Miseq short reads were scaffolded with long PacBio reads, and then the resultant scaffolds were gap-filled with PBJelly [20]. The genome assembly was improved by Pilon [21]. Besides, the chromosomal replication origin (*ori*C) was predicted by OriFinder [22]. The circular chromosome was subsequently confirmed by using PCR amplification and DNA sequencing of the upstream and downstream junctions of the *ori*C site.

The RJF293 genome sequence was annotated by the NCBI Prokaryote Genome Annotation Pipeline (PGAP) version 2.0 [23]. Putative virulence factors were predicted by VRprofile with the BLASTp-based *Ha*-value >0.64 [24], which collected 2454 virulence factors from the VFDB database [25]. Unusual chromosomal regions with putative foreign origins were identified by VRprofile with default parameters, including prophage, integrative and conjugative element (ICE) and genomic islands [25]. The type IV and VI secretion systems were also predicted by VRprofile. CRISPR (Clustered Regularly Interspaced Short Palindromic Repeats) arrays were identified by PILERCR [26].

### Genomic comparative analysis

Genome-wide single nucleotide polymorphism (SNP) calling and phylogenetic analysis was performed by using kSNP v3 [27-29]. The 106 completely sequenced *K. pneumoniae* genomes, including RJF293 sequenced in this study, were downloaded from NCBI Genome (Supplementary Table S3). The phylogeny scheme was generated from the 429,267 kSNP3-detected SNP sites for all the chromosome sequences with *k* = 21, as determined by Kchooser [27]. A parsimony tree was generated by kSNP3 based on an extended majority rule consensus of the equally most parsimonious trees from a sample of 100 trees [27]. The tree was displayed with iTOL with midpoint rooting [30].

Genome sequence comparisons between RJF293 and the other seven completely sequenced hvKP strains (Supplementary Table S4) were performed by mGenomeSubtractor [31]. All the annotated RJF293 protein-coding genes (served as the query) were examined by mGenomeSubtractor-facilitated BLASTn searches, using an *H*-value cut-off for conserved genes, against the other hvKP genomes (served as subject). The *H*-value (0 ≤ *H*-value ≤ 1.0) reflects the degree of similarity in terms of the length of match and the degree of identity at the nucleotide level between the matching gene in the subject genome and the query gene examined [31]. *Klebsiella pneumoniae* strains were defined as hvKP if they exhibited the hypermucoviscous phenotype and caused severe and metastatic infection [3].

### Detection of ICE and prophage excision

The excision of prophage or ICE from the chromosome was detected using PCR assays. Primers were picked upstream of the *attL* (P1) and downstream of the *attR* (P4) sites, with distance of 100–1000 bp (Supplementary Table S2). No amplicon can be obtained with this pair of primers if the ICE or prophage remains integrated in the chromosome, as the size of ICE or prophage is outside the range of DNA polymerase capability. The *attB* site after excision of the ICE or prophage is normally smaller than 100bp in size. When the ICE or prophage identified can excise from the chromosome, an amplicon with the expected size can be observed using PCR amplification (Supplementary Figure S1).

### Nucleotide sequence accession numbers

The genome sequence of *Klebsiella pneumoniae* RJF293 has been submitted to GenBank under accession numbers CP014008 (RJF293 chromosome) and CP014009 (plasmid pRJF293).

## Results

### Collection and clinical characteristics of hypermucoviscous K. pneumoniae isolates

From September 2014 to March 2016, a total of 872 *K. pneumoniae* episodes were collected by the Clinical Microbiology Laboratory of the Ruijin Hospital. A total of 128 *K. pneumoniae* isolates (128/872, 14.67%) exhibited the hypermucoviscous phenotype (positive in string test), and the highest prevalence of 44.1% was detected in pus samples. The mean age of the patients was 62.2 ± 14.5 years. Ninety-one patients (91/128, 71.09%) were males, and 37 (37/128, 28.90%) were females. These clinical specimens were collected from respiratory samples (90/128, 70.3%), abscess samples (15/128, 11.7%), blood samples (5/128, 3.9%), abdominal drainage samples (5/128, 3.9%), urine samples (4/128, 3.1%), secretion samples (4/128, 3.1%), bile samples (3/128, 1.6%) and pleural effusion samples (2/128, 1.6%). The hypermucoviscous isolates occurred commonly in patients with diabetes mellitus (28.9%), biliary tract disease (27.3%), or cancer/immunosuppression (44.5%), and 27.3% of patients had a history of abdominal surgery. Of the hypermucoviscous *K. pneumoniae* infections, 25% led to sepsis, and 7% of patients developed septic shock and died during hospitalization.

### Microbiological characteristics of K. pneumoniae isolates with ultra-long mucoid string

During the string test, 20 hypermucoviscous *K. pneumoniae* isolates exhibited an ultra-long viscous string (> 20 mm; RJF293 as representative in the Supplementary Figure S2). They included 4 isolates from blood samples, 9 from the respiratory tract, 4 from abscesses, 1 from abdominal drainage, and 2 from other samples (Table 1). PFGE results showed that these 20 isolates had 18 distinct patterns (Supplementary Figure S3A). For capsular serotypes, the K1 (*n* = 7) and K2 (*n* = 6) serotypes were predominant (Table 1). There were a total of 9 MLST types in these 20 isolates, including two new types (Table 1). The K2 serotype isolates belonged to three different sequence types, ST374, ST86 and ST375, whereas, 71.4% of K1 serotype isolates belonged to ST23.

**Table 1.** The capsular serotype, MLST type and distribution of ten virulence factor genes in the 20 hypermucoviscous *K. pneumoniae* isolates with ultra-long viscous string (>20 mm)^a^.

Isolate	Patient Gender	Patient Age	Collection Date	Source	Department	MLST ^b^	CPS	*magA*	*allS*	*rmpA*	*mrkD*	*kfuBC*	*cf29a*	*fimH*	*uge*	*wabG*	*ureA*
RJA360	m	77	Sept., 2014	sputum	neurology	23	K1	+	+	+	+	+	+	+	+	+	+
RJF67-2	m	63	Oct., 2014	blood	EICU	23	K1	+	+	+	+	+		+	+	+	+
RJA2570	f	75	Dec., 2014	abscess	trauma surgery	23	K1	+	+	+	+	+	+	+	+	+	+
RJF999	m	46	Jan., 2015	blood	ICU	23	K1	+	+	+	+	+		+	+	+	+
RJA166	m	47	Apr., 2015	sputum	cardiac surgery	23	K1	+	+	+	+	+		+	+	+	+
RJF271	m	54	Apr., 2014	abscess	emergency	680	K1	+	+	+	+	+	+	+	+	+	+
RJA277	m	55	Nov., 2014	abscess	intervention	2846	K1	+		+	+		+	+	+	+	+
RJA304	f	64	Sept., 2014	sputum	dermatology	86	K2			+	+			+	+	+	+
RJB442	f	71	Oct., 2014	urine	nephrology	86	K2			+	+			+	+	+	+
RJF293	m	54	Sept., 2014	blood	ICU	374	K2		+	+	+	+		+	+	+	+
RJF294	m	54	Sept., 2014	blood	ICU	374	K2		+	+	+	+		+	+	+	+
RJA898	m	54	Sept., 2014	sputum	ICU	374	K2		+	+	+	+		+	+	+	+
RJA2225	m	61	Nov., 2014	abscess	general surgery	375	K2			+	+			+	+	+	+
RJA1385	m	38	Feb., 2015	drainage	EICU	11	Not tested				+			+	+	+	+
RJA1253	m	59	Mar., 2014	hydrothorax	thoracic surgery	412	Not tested			+	+			+	+	+	+
RJA1657	f	71	Sept., 2014	sputum	thoracic surgery	412	Not tested			+	+			+	+	+	+
RJA565	f	43	Sept., 2014	sputum	hematology	412	Not tested			+	+			+	+	+	+
RJA1547	f	74	Nov., 2014	bile	transplantation	412	Not tested			+	+			+	+	+	+
RJA1504	f	44	Dec., 2014	sputum	respiratory	412	Not tested			+	+			+	+	+	+
RJA1887	m	74	Sept., 2014	sputum	respiratory	2845	No tested			+	+	+		+	+	+	+

a Virulence factor genes of *K. pneumoniae*: *magA*, coding for polysaccharides polymerase specific for *K. pneumoniae* serotype K1; *allS*, activator of the allantoin regulon;*rmpA*, transcriptional activator of *cps* gene transcription; *mrkD*, adhesin subunit of type 3 fimbriae; *kfuBC*, iron transport and phosphotransferase protein; c*f29a*, CF504 protein precursor; *fimH*, minor adhesin subunit of type 1; *uge*, uridinediphosphate galacturonate 4-epimerase; *wabG*, glucosyltransferase; *ureA*, urease subunit gamma; fimbriae.

b Multilocus Sequence Typing (MLST) was determined by BIGSdb (http://bigsdb.pasteur.fr/klebsiella/).

In order to determine whether the above-identified clones differ by their virulence potential, the presence of 10 reported genetic factors implicated in *K. pneumoniae* virulence was examined by PCR (Table 1). All the 20 hypermucoviscous *K. pneumoniae* isolates harbored the *mrkD*, *fimH*, *uge*, *wabG* and *ureA* genes. *rmpA*, the gene responsible for the hypermucoviscous phenotype was absent in one isolate. Nearly three-fourths of these isolates carried more than 7 virulence genes.

In addition, antimicrobial susceptibility testing showed that all 20 hypermucoviscous *K. pneumoniae* isolates were resistance to ampicillin but susceptible to the majority of the other antimicrobial agents tested, including cephalosporins, β-lactam/β-lactamase inhibitor and carbapenems (Supplementary Table S5). Namely, the percentage of strains susceptible to ampicillin/sulbactam, cefazolin, ceftazidime, ciprofloxacin, amikacin, and imipenem was 90%, 90%, 95%, 95%, 100% and 100%, respectively.

### Clinical characteristics of hvKP isolate RJF293 with serotype K2 and ST374

Our attention was drawn to a fatal septic shock patient with a bloodstream infection caused by a hypermucoviscous *K. pneumoniae* strain. The patient was a 64-year-old male who was admitted to surgery at the Ruijin Hospital due to gastric stump cancer on March 2014. He received total gastrectomy, complicated with postoperative bleeding, and then underwent hemostatic laparotomy the next day. With high temperature and breathing difficulty, the patient developed septic shock due to the abdominal infection and was subsequently transferred to the ICU 20 days later and received abdominal drainage. Cefuroxime, meropenem and ceftazidime were administrated to this patient for anti-infection treatment, but unfortunately the infection persisted and this patient died 5 months after entrance into the ICU.

Routine microbiological cultures of sputum, drainage and blood samples were collected from this patient. Four *K. pneumoniae* isolates were identified, including RJA726 (not included in the previous 20 hypermucoviscous strains due to its isolation period) isolated from the abdominal drainage in April, RJF293 and RJF294 from the blood in September, and RJA898 from the sputum in September. All four *K. pneumoniae* isolates were characterized as hypermucoviscous by string test (RJF293 as example, Supplementary Figure S2). These 4 isolates had the same PFGE pattern, capsular serotype (K2), MLST type (ST374) and antimicrobial susceptibility testing results (Supplementary FigureS3B, Table1 and Supplementary Table S5). This evidence suggested toward the metastasis of the same hypermucoviscous *K. pneumoniae* strain in this patient. RJF293 collected from the bloodstream was selected as the representative for further virulence assays, genome sequencing, and comparative analysis.

### Mouse lethality assay exhibited hypervirulence of RJF293

**Figure 1.**
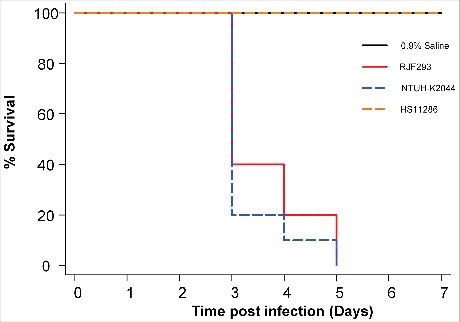
Kaplan-Meier survival curves for *K. pneumoniae* RJF293 infected mice. Mice were infected with 10^3^ CFU of different *K. pneumoniae* strains intraperitoneally. The previously reported hvKP strain NTUH-K2044 (ST23, K1 serotype), cKP strain HS11286 (ST11, KL103 serotype) and saline were applied as the controls. RJF293 showed virulence not statistically significant from that of NTUH-K2044 (*p* > 0.6, by log-rank test). No death of mice in the HS11286 or saline groups was observed during seven days.

The susceptibility of BALB/c mice to the *K. pneumoniae* RJF293 strain was examined to study its pathogenicity. Five-week-old mice in healthy condition were infected with a serial dilution of bacteria, and the survival was recorded every 24 hours, within 7 days post-infection (Fig. 1). RJF293 showed an LD50 of 1.5 × 10^2^ CFU in the BALB/c mice (calculated by Reed-Muench method). For the survival analysis, 10^3^ CFU of different strains as well as only saline were injected into the mice intraperitoneally. The Kaplan-Meier survival estimate revealed that RJF293 showed virulence similar to that of hvKP strain NTUH-K2044 (*p* = 0.461, by log-rank test). The ST11 cKP strain HS11286 did not cause mortality within the observation duration. These results showed that the ST374 serotype K2 strain RJF293 was equivalently virulent to the well-documented ST23 serotype K1 hvKP strain NTUH-K2044 in the mouse infection model.

### RJF293 genome analysis

Since no K2 ST374 *K. pneumoniae* strain had yet been completely sequenced, we decided to explore the virulence factors of RJF293 via WGS. Our analysis showed that the RJF293 genome consists of a circular chromosome of 5,226,330 base pairs with average GC content of 57.5%, and one circular plasmid with 224,263 bases with average GC content of 50.1%. The RJF293 chromosome contains 4,995 annotated protein-coding sequences (CDSs) (Supplementary Table S4). The size of the plasmid was confirmed using S1-nuclease treatment followed by PFGE (Supplementary Figure S4).

**Figure 2.**
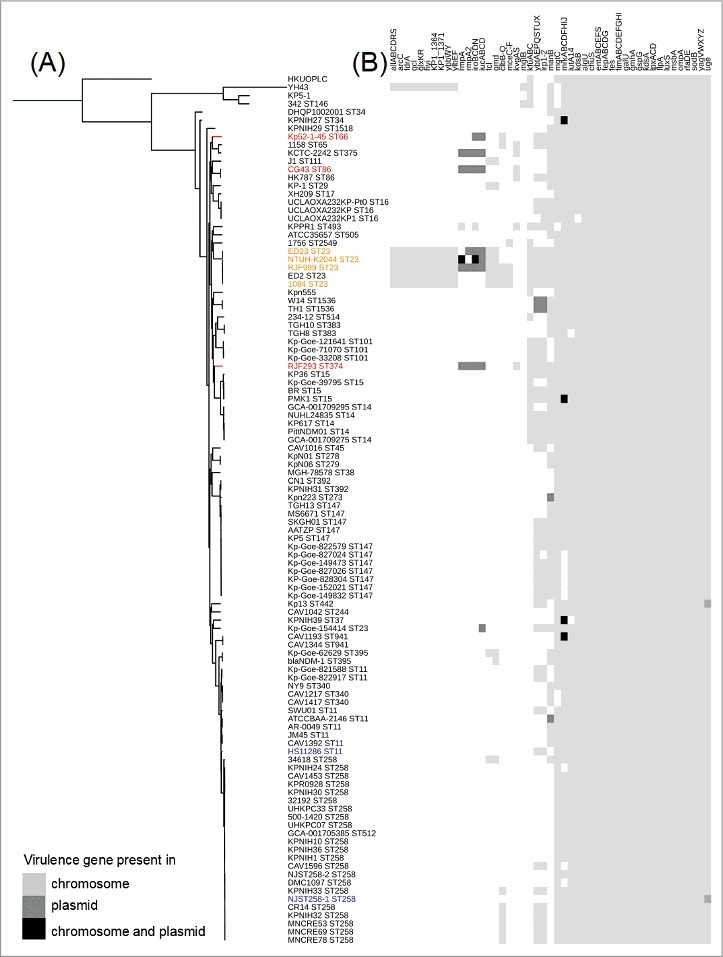
Putative virulence genes (gene clusters) detected among the 106 completely sequenced *K. pneumoniae* genomes (Supplementary Table S3). (A) A parsimony tree generated from 429,267 SNPs using kSNP3 for the 106 completely sequenced *K. pneumoniae* chromosomes and displayed by iTOL with midpoint rooting. The hvKP and cKP isolates listed in Supplementary Table S4 are highlighted by color (red or orange, hvKP; blue, cKP). **(B)** The virulence genes predicted in the RJF293 genome are listed as an example in Supplementary Table S5. The presence of genes in the chromosome and/or plasmid is indicated in different gray scales.

The RJF293 strain exhibits a distinct genetic background from those of the other sequenced K2 and K1 serotype hvKP strains, as supported by both phylogenetic analysis and genome alignments. Whole genome SNP-based phylogenetic analysis showed that the ST374 RJF293 was failed to be grouped with any other K2 serotype hvKP strains (ST66 or ST86), also distant from the clade grouped by the ST23 hvKP strains (Fig. 2A). In addition, when all 4,995 CDSs of RJF293 were analyzed for the presence of homologs against seven completely sequenced hvKP chromosomes (Supplementary Figure S5), only 88% (4,412/4,995) of homologous CDSs were found across all eight hvKP genomes being compared (BLASTn-based *H*-value > 0.81) [31].

A total of 69 putative virulence genes were detected on the RJF293 chromosome (Supplementary Table S6), including gene clusters coding for enterobactin synthesis (*fep*, *ent*), pilus (*yag*), type 3 fimbriae (*mrk*), lipopolysaccharide synthesis (*lpx*) and iron ABC transporter (*kfu*). These RJF293 virulence genes show a unique presence/absence pattern (Fig. 2B). Compared with the ST23 K1 serotype hvKP strains, the two-component regulatory system KvgAS is found to be present in only RJF293 (K2, ST374) and another five genomes of *K. pneumoniae* K2 strains*.* Besides, 8 virulence genes (clusters), including *glxKR, ybbWY, allABCDRS, ylbEF, hyi, arcC, fdrA, KP1_1364* and *KP1_1371*, were detected only in the five ST23 K1 serotype hvKP strains and another strain (YH43 with a novel sequence type and absence of capsular serotype information). The *cps* gene clusters of K1 and K2 *K. pneumoniae* strains have conserved regions at the 5′ and 3′ extremities, while the variable region is located between *wzb* and *wcaJ* (Supplementary Figure S6). RJF293 showed 100% identity to the previously reported K2 strain CG43 (ST86) in the *cps* gene cluster region, though with different MLST patterns [32]. The LPS cluster of RJF293 was characterized as O1, which is the most prevalent serotype in *K. pneumoniae* strains, and an IS*102* element was located upstream of the RJF293 LPS cluster (Supplementary Figure S7) [33].

One likely intact prophage was identified within the 40-kb region (GI5) specific to the RJF293 chromosome. The corresponding excision form of this prophage was identified by a PCR assay (Supplementary Figure S8) and the sequence of *attB* site after excision was determined to be TTGAACAT. This prophage coded for the prophage core component proteins, including the coat, plate, portal, tail, and tail fiber proteins and integrases. Interestingly, it also contains a 3-kb variable region coding for seven RJF293-specific CDSs with unknown function. Six other large unusual GI-like regions (>10 kb) were detected on the RJF293 chromosome (Table 2 and Supplementary Figure S5), which contained G+C contents lower than the genomic G+C content. For example, the 20-kb region GI8 includes a *kvgAS* locus (*RJF2_RS21335-RJF2_RS21340*) and three genes coding for fimbrial (or chaperone) proteins.

**Table 2.** Large genomic island-like regions (>10 kb) identified on the *K. pneumoniae* RJF293 chromosome.

Region	Coordinates [CDS]	Length (kb)	G+C%^a^	Features
GI1	406,963-417,086 [RJF2_RS01995-02030]	10.1	44.6	Fimbrial assembly protein
GI2	564,594-609,498 [RJF2_RS02780-03005]	44.9	47.0	Insertion site: *tRNA^Leu^*; integrase (RJF2_RS02780)
GI3	1,066,669-1,077,261 [RJF2_RS05225-05250]	10.6	44.7	Radical SAM protein (RJF2_RS05240); integrase (RJF2_RS05250)
GI4	2,033,163-2,043,180 [RJF2_RS09775-09830]	10.0	50.2	
GI5 (prophage)	2,364,000-2,417,629 [RJF2_RS11405-11765]	53.6	51.7	Intact prophage; integrase (RJF2_RS11765)
GI6 (ICE)	3,380,804-3,439,781 [RJF2_RS16675-16850]	59.0	53.1	Yersiniabactin synthesis, Type IV secretion system. Insertion site:tRNA^Asn^;integrase (RJF2_RS16670)
GI7 (T6SS)	3,762,797-3,804,165 [RJF2_RS18280-18450]	41.4	50.7	Type VI secretion system (T6SS-3)
GI8	4,379,704-4,400,294 [RJF2_RS21295-21365]	20.6	46.8	Two-component regulatory system KvgAS; Fimbrial assembly protein

a The G+C content of the RJF293 chromosome is 57.5%.

The type VI secretion system (T6SS) has been shown to be present in several Gram-negative species [34]. There are three different type VI secretion system loci on the RJF293 chromosome (T6SS-1, *RJF2_RS10835-10935*; T6SS-2, *RJF2_RS15665-15720*; T6SS-3, *RJF2_RS18290-18405*), among which T6SS-3 was located on the 41-kb island GI7. This T6SS locus is present in five out of the 105 other completely sequenced *K. pneumoniae* strains, including CAV1042, Kp_Goe_121641, Kp_Goe_71070, 234-12, and Kpn555. Its cognate effector (*RJF2_RS18365*) contains a type I dockerin repeat domain, which is the binding partner of the cohesin domain, and the cohesin-dockerin interaction is the crucial interaction for complex formation in the cellulosome. The finding of multiple T6SS loci is consistent to previous studies [35,36]. and it still remains to be uncovered in the further study whether the three T6SS loci of RJF293 can function independently or not.

The RJF293 chromosomally borne ICE (GI6), called ICE*Kpn*RJF293, is inserted into a *tRNA^Asn^* locus and contains a virulence factor gene cluster involved in the synthesis, regulation, and transport of the siderophore yersiniabactin (Supplementary Figure S9). This 59-kb ICE also codes for a conjugation transfer-associated type IV secretion system. The PCR assay showed that this ICE was able to site-specifically excise from the 3′-end of the *tRNA^Asn^* gene on the RJF293 chromosome (Supplementary Figure S10). ICE*Kpn*RJF293 is highly syntenic to ICE*Kp*1 in the liver abscess-causing hvKP strain NTUH-K2044 (ST23, K1 serotype) but lacks a virulence-associated region with an *iroBCDN* gene cluster responsible for salmochelin biosynthesis (Supplementary Figure S9) [37]. Notably, ICE*Kpn*RJF293 contains a 10-kb strain-unique region in the tRNA-distal end, which codes for a restriction-modification system, an ABC transporter, a hypothetical protein and two transposases (Supplementary Figure S9).

Two CRISPR arrays were also detected: one is located from 2,991,230 to 2,991,753bp and contains 8 spacers; another is from 3,001,570 to 3,001,903bp and contains 5 spacers. The seven CRISPR-associated protein genes (type I-E) are located between the two CRISPR arrays, forming a sandwich structure like the experimentally verified system in *K. pneumoniae* NTUH-K2044 [38]. The DRs of the CRISPR arrays are conserved between RJF293 and NTUH-K2044, but the spacer sequences vary, revealing the difference in habitats of these two strains. In addition, the chromosome contains 12 annotated insertion sequence (IS) elements belonging to five families, i.e., IS*5* (*n* = 5), IS*3* (*n* = 4), IS*1* (*n* = 1), IS*481* (*n* = 1) and Tn*3* (*n* = 1).

### RJF293 carries a virulence plasmid, pRJF293

The 224-kb plasmid pRJF293 exhibits high sequence similarity to the reported 219-kb virulence plasmid pLVPK (NCBI accession no. NC_005249) in the hvKP strain CG43 (ST86, K2 serotype) (Supplementary Figure S11) [39]. It codes for 11 putative virulence genes (Supplementary Table S6)*,* including the mucoid phenotype regulator genes *rmpA* (*RJF2_RS26130*) and *rmpA2* (*RJF2_RS26605*)*,* the *iroBCDN* gene clusters involved in salmochelin biosynthesis (*RJF2_RS26145– RJF2_RS26160*), and the *iucABCD/iutA* gene cluster related to aerobactin biosynthesis (*RJF2_RS26550- RJF2_RS26570*). These virulence determinants are encoded on pRJF293 or similar virulence plasmids reported in *K. pneumoniae* (120-230 kb in size), which has been identified as restricted to hvKP isolates, including RJF293, CG43, KCTC2242, RJF999, ED23, Kp52.145 and NTUH-K2044 (Fig. 2B). We also annotated 14 IS elements in pRJF293. They belong to seven families, IS*5* (*n* = 5), IS*1* (*n* = 3), IS*3* (*n* = 2), Tn*3* (*n* = 2), IS*21* (*n* = 1), and IS*66* (*n* = 1). Additionally, compared to plasmid pLVPK of the ST86 K2 serotype hvKP strain CG43, pRJF293 carries a novel 5.7-kb transposon (region 184,481..190,190 bp) [39]. This transposon is composed of two IS*102* elements at the ends, which are related to the IS*903* group of the IS*5* family. It also contains five annotated CDSs coding for putative relaxase and five putative proteins that may confer DNA conjugative transfer. This pRJF293-carrying transposon is only present in the virulence plasmid of hvKP isolate RJF999 (ST23, K1 serotype, isolated in the same hospital as RJF293, GenBank accession no. CP014010.1), but absent from the other 104 completely sequenced *K. pneumoniae* genomes.

## Discussion

Recently hypervirulent *K. pneumoniae* variants have been emerging as a cause of severe hospital-acquired invasive infections in critical patients [1]. Increasing numbers of hvKP isolates being collected from blood samples of ICU patients in our study and in other clinical settings was reported [4,13]. The identification of multiple MLST allelic types and capsular serotypes reflects the genetic diversity of hypermucoviscous *K. pneumoniae* isolates. Among the 79 known capsular serotypes of *K. pneumoniae*, K1 and K2 have been shown to be the most prevalent in hvKP, and their association with invasive infection has been corroborated using murine models [6,40,41]. In practice, K2 hvKP shows high clinical significance due to its high pathogenicity and metastatic infection capability. In comparison to K1 hvKP, K2 hvKP shows high MLST diversity, calling for more genomic data for understanding its pathogenesis [6].

In our study, the K2 serotype ST374 strain RJF293 was collected from a human blood sample in an ICU patient. RJF293 exhibited three typical features of hvKP, showing the hypermucoviscous phenotype (viscous string of >20 mm in length) and causing severe and metastatic infection [3]. The mouse lethality assay also confirmed that RJF293 was as virulent as the ST23 K1 serotype strain NTUH-K2044. We examined the *K. pneumoniae* isolates collected in our ICU department. However, no isolates from other patients showed the same MLST or PFGE pattern as RJF293.

The existence of 47 virulence-associated genes (gene clusters) in the 106 *K. pneumoniae* strains with a complete genome sequence was determined in our study. Strains with serotype K1 and K2 showed a remarkable difference in the carriage of these gene/gene clusters, and the K1 strains carried significantly more of these genes (gene clusters) than the K2 strains. *allABCDR*, *arcC*, *fdrA*, *gcl*, *glxKR*, *hyi*, *KP1_1364*, *KP1_1371*, *ybbWY*, *ylbEF*, *fcl*, and *gmd* are present in only serotype K1 *K. pneumoniae* genomes. These genes have been reported to be contributive to bacterial virulence. For example, the allantoinase gene cluster *allABCDR* involved in allantoin metabolism has been shown to contribute to virulence in mice after gastrointestinal inoculation [42]. By mouse lethality assay, *K. pneumoniae* RJF293 was determined to have the same level of virulence as the K1 *K. pneumoniae* NTUH-K2044. This revealed that having more virulence-associated genes (gene clusters) is not equal to a higher level of virulence. The contribution of each known virulence determinant needs to be assessed quantitatively.

Different from the virulence determinants stated above, most of the genes/gene clusters located on plasmids, ICEs and other mobile genetic elements are shared by both K1 and K2 *K. pneumoniae*. These include the well-documented hypermucoviscous phenotype regulators *rmpA* and *rmpA2*, the aerobactin synthesis cluster *iucABCD*, the salmochelin synthesis cluster *iroBCDN* etc. The *rmpA* and *rmpA2* genes have both been shown to activate capsule production, exhibiting the hypermucoviscous phenotype [43,44]. Siderophore systems are considered integral to bacterial virulence, allowing bacteria to scavenge for iron from host transport proteins, thereby enhancing the ability to survive and replicate within the host [45]. The aerobactin has been shown to be the predominant siderophore in the hypervirulent *K. pneumoniae* [46]. The research conducted by Russo *et al* demonstrated that the contributions of different siderophores for *K. pneumoniae* growth and survival in human ascites fluid were not equivalent. Aerobactin showed relatively higher biological activity than yersiniabactin or salmochelin [47]. These genes (gene clusters) are usually carried by a virulence plasmid belonging to the IncHI1B group or an ICE belonging to the ICE*Kp1* family. The virulence plasmid (in this study, pRJF293) is normally 120 kb to 230 kb in size. It is majorly carried by *K. pneumoniae* strains with capsular serotype K1 or K2. However, very recently, the virulence plasmid was found in a K47 ST11 *K. pneumoniae* strain in China [48]. It was characterized as greatly contributive to the ST11 hvKP and might be a sign of the dissemination of virulence plasmids in the future. The ICE*Kp1* family is another group of mobile genetic elements shared by K1 and K2 *K. pneumoniae* strains. The cargo genes (gene clusters) on this ICE can code for yersiniabactin, salmochelin and colibactin. In fact, the ICE is widely distributed in *K. pneumoniae* (47 out of 106) with more capsular serotypes and MLST types, for example *K. pneumoniae* AATZP (K64, ST147) [49]. The virulence plasmid together with the ICE make up a platform for the intra- and even inter-species dissemination of virulence determinants.

In summary, we investigated the genetic background and microbiological features of a hypermucoviscous clinical isolate (RJF293) of *K. pneumoniae* collected from human blood. The metastatic infection case, string test, and mouse lethality assay confirmed the hypervirulence of this ST374 K2 serotype strain. Thus, the RJF293 genomics study reported here represents the first complete genome sequence of an ST374 *K. pneumoniae* strain with the K2 serotype, making a valuable addition to the growing list of diverse hvKP genomes for the scientific research community worldwide. Our results suggest that the ST374 K2 serotype hvKP case should not be underestimated and its clinical impact would be particularly challenging.

## Supplementary Material

1421894_supp.pdf
