# Supplementary material for: Whole-Genome-Sequencing characterization of bloodstream infection-causing hypervirulent Klebsiella pneumoniae of capsular serotype K2 and ST374
Source: Virulence. 2018 Feb 27;9(1):510–21. doi: 10.1080/21505594.2017.1421894 (PMC5955473; doi:10.1080/21505594.2017.1421894)
Supplement: 1421894_supp.pdf [file kvir-09-01-1421894-s001.pdf]

Whole-Genome-Sequencing characterization of bloodstream infection-causing hypervirulent *Klebsiella pneumoniae* of capsular serotype K2 and ST374

Xiaoli Wang[1,§], Yingzhou Xie[2,§], Gang Li [3,4], Jialin Liu[1], Xiaobin Li[2], Lijun Tian[1], Jingyong Sun[5], Hong-Yu Ou[2,*], Hongping Qu[1,*]

[1] Department of Critical Care Medicine, Ruijin Hospital, Shanghai Jiao Tong University School of Medicine, Shanghai, China

[2] State Key Laboratory of Microbial Metabolism, Joint International Laboratory on Metabolic & Developmental Sciences, School of Life Sciences & Biotechnology, Shanghai Jiao Tong University, Shanghai, China

[3] Department of Laboratory Medicine, Jinshan Hospital, Shanghai Medical College, Fudan University, Shanghai, China

[4] Department of Laboratory Medicine, Huashan Hospital, Shanghai Medical College, Fudan University, Shanghai, China

[5] Department of Clinical Microbiology, Ruijin Hospital, Shanghai Jiaotong University School of Medicine, Shanghai, China

*Correspondence and requests for materials should be addressed to H.Q. (hongpingqu0412@hotmail.com), H.Y.O (hyou@sjtu.edu.cn).

§ These authors contributed equally to this work.

Supplementary data

Table S1. Bacterial stains used in this study.

Table S2. Oligonucleotides used in this study.

Table S3. List of the 106 GenBank-archived completely sequenced *K. pneumoniae* strains under analysis.

Table S4. General features of the *K. pneumoniae* RJF293 genome and others be compared.

Table S5. Antimicrobial susceptibility tests of the hypermucoviscous *K. pneumoniae* isolates with ultra-long mucoid string (>20 mm).

Table S6. Putative virulence factor genes detected in the genome of hypervirulence *K. pneumoniae* of RJF293.

Table S7. List of the putative virulence factor detected in the 106 *K. pneumoniae* complete genome sequences.

Figure S1. Detection of the ICE or prophage excision by PCR.

Figure S2. Positive hypermucoviscosity phenotype of *K. pneumoniae* RJF293 by string test.

Figure S3. Gel image of PFGE for hypermucoviscous *K. pneumoniae* clinical isolates.

Figure S4. Gel image of S1-PFGE for *K. pneumoniae* RJF293.

Figure S5. The mGenomeSubtractor-based *in silico* subtractive hybridization of the *K. pneumoniae* RJF293 genome against genomes of other seven completely sequenced hvKP isolates.

Figure S6. Alignment maps of the five representative *K. pneumoniae* CPS gene clusters*.*

Figure S7 Alignments between the O1 LPS gene cluster in *K. pneumoniae* NTUH-K2044, RJF293 and Friedlander 204.

Figure S8. Excision of PHAGE_*Kpn*RJF293 from the RJF293 chromosome.

Figure S9. Sequence alignments between four ICE*Kp*1 family ICEs.

Figure S10. Site-specifically excision of ICE*Kpn*RJF293 from the 3'-end of the $tRNA^{Asn}$ gene on the RJF293 chromosome.

Figure S11. Diagram of the plasmid pRJF293 and the sequence alignments to four other completely sequenced *K. pneumoniae* virulence plasmids.

Table S1. Bacterial stains used in this study.

Strain	Genotype	Key feature	Reference
K. pneumoniae			
RJA360	ST23, Capsular serotype K1	Isolated from sputum sample of patient in Neurology Department.	Lab archive
RJF67-2	ST23, Capsular serotype K1	Isolated from blood sample of patient in EICU.	Lab archive
RJA2570	ST23, Capsular serotype K1	Isolated from abscess sample of patient underwent trauma surgery.	Lab archive
RJF999	ST23, Capsular serotype K1	Isolated from blood sample of patient in ICU.	Lab archive
RJA166	ST23, Capsular serotype K1	Isolated from sputum sample of patient underwent cardiac surgery	Lab archive
RJF271	ST680, Capsular serotype K1	Isolated from abscess sample of patient from Emergency.	Lab archive
RJA277	Non-typable MLST, Capsular serotype K1	Isolated from abscess sample of patient from Inrervention Department.	Lab archive
RJA304	ST86, Capsular serotype K2	Isolated from sputum sample of patient in Dermatology Department.	Lab archive
RJB442	ST86, Capsular serotype K2	Isolated from urine sample of patient in Nephrology Department.	Lab archive
RJF293	ST374, Capsular serotype K2	Isolated from blood sample of patient in ICU.	Lab archive
RJF294	ST374, Capsular serotype K2	Isolated from blood sample of patient in ICU.	Lab archive
RJA898	ST374, Capsular serotype K2	Isolated from sputum sample of patient in ICU.	Lab archive
RJA2225	ST375, Capsular serotype K2	Isolated from abscess sample of patient underwent general surgery.	Lab archive
RJA1385	ST11, Non-typable capsular serotype	Isolated from drainage sample of patient in EICU.	Lab archive
RJA1253	ST412, Non-typable capsular serotype	Isolated from hydrothorax sample of patient underwent thoracic surgery.	Lab archive
RJA1657	ST412, Non-typable capsular serotype	Isolated from sputum sample of patient underwent thoracic surgery.	Lab archive
RJA565	ST412, Non-typable capsular serotype	Isolated from sputum sample of patient in Hematology Department.	Lab archive
RJA1547	ST412, Non-typable capsular serotype	Isolated from bile sample of patient underwent transplantation.	Lab archive
RJA1504	ST412, Non-typable capsular serotype	Isolated from sputum sample of patient in Respiratory Department.	Lab archive
RJA1887	Non-tyable MLST and capsular serotype	Isolated from sputum sample of patient in Respiratory Department.	Lab archive
RJA726	ST374, Capsular serotype K2	Isolated from drainage sample of patient in ICU	Lab archive
HS11286	ST11, KL103[a]	Carbapenemase producing, low virulence; Control for the virulence determination.	Lab archive
NTUH-K2044	ST23, serotype K1	Characterized as hypervirulence; Control for the virulence determination.	Wu, *et al.*[1]

[a]The K-locus was typed by using the tool Kaptive, based on the whole genome sequence of *K. pneumoniae* HS11286.[2]

Table S2. Oligonucleotides used in this study. F indicates the forward primer and R indicates the reverse primer

Primer name	Sequence (5'-3')
MLST	
rpoB-F	GGCGAAATGGCWGAGAACCA
rpoB-R	GAGTCTTCGAAGTTGTAACC
gapA-F	TGAAATATGACTCCACTCACGG
gapA-R	CTTCAGAAGCGGCTTTGATGGCTT
mdh-F	CCCAACTCGCTTCAGGTTCAG
mdh-R	CCGTTTTTCCCCAGCAGCAG
pgi1-F	GAGAAAAACCTGCCTGTACTGCTGGC
pgi1-R	CGCGCCACGCTTTATAGCGGTTAAT
pgi2-F	CTGCTGGCGCTGATCGGCAT
pgi2-R	TTATAGCGGTTAATCAGGCCGT
phoE-F	ACCTACCGCAACACCGACTTCTTCGG
phoE-R	TGATCAGAACTGGTAGGTGAT
infB1-F	CTCGCTGCTGGACTATATTCG
infB1-R	CGCTTTCAGCTCAAGAACTTC
infB2-F	ACTAAGGTTGCCTCCGGCGAAGC
tonB-F	CTTTATACCTCGGTACATCAGGTT
tonB-R	ATTCGCCGGCTGRGCRGAGAG
Capsular serotype	
wzx_K1-F	GTAGGTATTGCAAGCCATGC
wzx_K1-R	GCCCAGGTTAATGAATCCGT
wzy_K1-F	GGTGCTCTTTACATCATTGC
wzy_K1-R	GCAATGGCCATTTGCGTTAG
wzx_K2-F	GGAGCCATTTGAATTCGGTG
wzx_K2-R	TCCCTAGCACTGGCTTAAGT
wzy_K2-F	GGATTATGACAGCCTCTCCT
wzy_K2-R	CGACTTGGTCCCAACAGTTT
Ten known virulence factor genes	
magA-F	GTAGGTATTGCAAGCCATGC
magA-R	GCCCAGGTTAATGAATCCGT
rmpA-F	GCAGTTAACTGGACTACCTCTG
rmpA-R	GTTTACAATTCGGCTAACATTTTTCTTTAAG
allS-F	TCTGATTTAACCCACATT
allS-R	CCGTTAGGCAATCCAGAC
mrkD-F	TATTGGCTTAATGGCGCTGG
mrkD-R	TAATCGTACGTCAGGTTAAAGACC
kfuBC-F	GAAGTGACGCTGTTTCTGGC
kfuBC-R	TTTCGTGTGGCCAGTGACTC
fimH-F	GCTCTGGCCGATACCACCACGG
fimH-R	GCGAAGTAACGTGCCTGGAACGG
uge-F	GATCATCCGGTCTCCCTGTA
uge-R	TCTTCACGCCTTCCTTCACT

wabG-F	CGGACTGGCAGATCCATATC
wabG-R	ACCATCGGCCATTTGATAGA
ureA-F	GCTGACTTAAGAGAACGTTATG
ureA-R	GATCATGGCGCTACCTCA
ICE*Kpn*RJF293 & PHAGE_*Kpn*RJF293	
ICE293-P1	GGTGACGTTCAAGAGAGACC
ICE293-P4	GTGAATTCATCCTACTGGC
PHG293-P1	AAGCCGAGAAACAACGGCAC
PHG293-P4	GATCCGGTTCAGCATACGGT

[a] The PCR primers were also used for DNA sequencing, except for the gene *infB*, for which the primer *infB*2-F was used instead of the forward PCR primer, and for *pgi*, for which the primers *pgi*2-F and *pgi2*-R are used.

Table S3. List of the 106 GenBank-archived completely sequenced *K. pneumoniae* strains under analysis

Strain	MLST type [a]	Accession no.of chromosome genome	Strain	MLST type [a]	Accession no.of chromosome genome
1084	ST23	NC_018522	Kp-Goe-62629	ST395	CP018364
1158	ST65	NZ_CP006722	Kp-Goe-71070	ST101	CP018450
1756	ST2549	CP019219	Kp-Goe-821588	ST11	CP018692
234-12	ST514	NZ_CP011313	Kp-Goe-822579	ST147	CP018140
32192	ST258	NZ_CP010361	Kp-Goe-822917	ST11	CP018438
342	ST146	NC_011283	Kp-Goe-827024	ST147	CP018701
34618	ST258	NZ_CP010392	Kp-Goe-827026	ST147	CP018707
500-1420	ST258	NZ_CP011980	KP-Goe-828304	ST147	CP018719
AATZP	ST147	CP014755	KpN01	ST278	NZ_CP012987
AR-0049	ST11	CP018816	KpN06	ST279	NZ_CP012992
ATCC35657	ST505	CP015134	Kpn223	ST273	CP015025
BAA-2146	ST11	NZ_CP006659	Kpn555	not defined	CP015130
blaNDM-1	ST395	NZ_CP009114	KPNIH1	ST258	NZ_CP008827
BR	ST15	CP015990	KPNIH10	ST258	NZ_CP007727
CAV1016	ST45	CP017934	KPNIH24	ST258	NZ_CP008797
CAV1042	ST244	CP018671	KPNIH27	ST34	NZ_CP007731
CAV1193	ST941	NZ_CP013322	KPNIH29	ST1518	NZ_CP009863
CAV1217	ST340	CP018676	KPNIH30	ST258	NZ_CP009872
CAV1344	ST941	NZ_CP011624	KPNIH31	ST392	NZ_CP009876
CAV1392	ST11	NZ_CP011578	KPNIH32	ST258	NZ_CP009775
CAV1417	ST340	CP018352	KPNIH33	ST258	NZ_CP009771
CAV1453	ST258	CP018356	KPNIH36	ST258	CP014647
CAV1596	ST258	NZ_CP011647	KPNIH39	ST37	CP014762
CG43	ST86	NC_022566	KPPR1	ST493	NZ_CP009208
CN1	ST392	CP015382	KPR0928	ST258	NZ_CP008831
CR14	ST258	CP015392	MGH-78578	ST38	NC_009648
DHQP1002001	ST34	CP016811	MNCRE53	ST258	CP018437
DMC1097	ST258	NZ_CP011976	MNCRE69	ST258	CP018427
ED2	ST23	CP016813	MNCRE78	ST258	CP018428
ED23	ST23	CP016814	MS6671	ST147	NZ_LN824133
GCA-001705385	ST512	CP015822	NJST258-1	ST258	NZ_CP006923
GCA-001709275	ST14	CP016923	NJST258-2	ST258	NZ_CP006918
GCA-001709295	ST14	CP016926	NTUH-K2044	ST23	NC_012731
HK787	ST86	NZ_CP006738	NUHL24835	ST14	NZ_CP014004
HKUOPLC	not defined	NZ_CP012300	NY9	ST340	CP015385
HS11286	ST11	NC_016845	PittNDM01	ST14	NZ_CP006798
J1	ST111	NZ_CP013711	PMK1	ST15	NZ_CP008929
JM45	ST11	NC_022082	RJF293	ST374	NZ_CP014008
KCTC-2242	ST375	NC_017540	RJF999	ST23	NZ_CP014010
KP-1	ST29	NZ_CP012883	SKGH01	ST147	CP015500

Kp13	ST442	NZ_CP003999	SWU01	ST11	CP018454
KP36	ST15	CP017385	TGH10	ST383	NZ_CP012744
KP5	ST147	CP012426	TGH13	ST147	CP012745
KP5-1	not defined	CP008700	TGH8	ST383	NZ_CP012743
Kp52.145	ST66	NZ_FO834906	TH1	ST1536	CP016159
KP617	ST14	NZ_CP012753	UCLAOXA232KP	ST16	CP012561
Kp-Goe-121641	ST101	CP018735	UCLAOXA232KP1	ST16	CP012568
Kp-Goe-149473	ST147	CP018686	UCLAOXA232KP-Pt0	ST16	CP012560
Kp-Goe-149832	ST147	CP018695	UHKPC07	ST258	NZ_CP011985
Kp-Goe-152021	ST147	CP018713	UHKPC33	ST258	NZ_CP011989
Kp-Goe-154414	ST23	CP018337	W14	ST1536	CP015753
Kp-Goe-33208	ST101	CP018447	XH209	ST17	NZ_CP009461
Kp-Goe-39795	ST15	CP018458	YH43	not defined	NZ_AP014950

[a] Multilocus Sequence Typing (MLST) was determined by BIGSdb.[3]

Table S4. General features of the *K. pneumoniae* RJF293 genome and others be compared [a]

Parameter	RJF293	CG43	Kp52.145	KPPR1	NTUH-K2044	1084	ED23	RJF999	HS11286	NJST258-1
hvKP/cKP [b]	hvKP	hvKP	hvKP	hvKP	hvKP	hvKP	hvKP	hvKP	cKP, *bla*$_{KPC-2}$	cKP, *bla*$_{KPC-3}$
Isolation source	Human, blood	Human, Liver pus	-	-	Human, Liver pus	-	Human, blood	Human, blood	Human, sputum	Human, urine
Capsular serotype	K2	K2	K2	-	K1	K1	K1	K1	KL103 [f]	-
ST no.	374	86	66	493	23	23	23	23	11	258
Chromosome										
size (bp)	5,226,330	5,166,857	5,438,894	5,374,834	5,248,520	5,386,705	5,374,626	5,461,919	5,333,942	5,263,229
No. of annotated CDSs	4,995	4,979	5,230	5,136	5,021	5,109	5,172	5,211	5,316	5,192
No. of virulence factors [c]	32	30	29	29	42	39	42	43	25	24
No. of putative prophages	1	2	4	2	1	1	3	4	7	9
No. of putative ICEs	1	0	1	1	2	1	1	2	2	0
No. of putative T4SS	1	0	1	1	2	2	2	2	2	1
No. of putative T6SS [d]	3	2	3	2	2	2	2	2	2	2
No. of putative CRISPR arrays [e]	2	0	2	2	2	2	1	2	0	0
No. of plasmids	1	1	2	0	1	0	1	1	6	5

[a] ST, sequence type; CDS, protein coding sequences; ICE, integrative and conjugative elements; T4SS, type IV secretion system; T6SS, type VI secretion system; CRISPR, clustered regularly interspaced short palindromic repeats; hvKP, hypervirulent *Klebsiella pneumoniae;* MDR, multiple drug resistance.

[b] The classifications of hvKP or cKP strains were taken from the corresponding publications. In general, they exhibited three typical features, including (i) causing severe infection, (ii) metastatic spread of infection, and (iii) hypermucoviscous phenotype.[4]

[c] The 45 *K. pneumoniae* virulence genes (clusters) under analysis are shown in Figure 5. The description of virulence genes are also listed in Table S4.

[d] The putative T6SS gene clusters with was detected by VRprofile (http://bioinfo-mml.sjtu.edu.cn/VRprofile/).[5]

[e] The putative CRISPR arrays was identified by PILERCR (http://www.drive5.com/pilercr/).[6]

[f] The K-locus was typed by using the tool Kaptive, based on the whole genome sequence of *K. pneumoniae* HS11286.[2]

Table S5. Antimicrobial susceptibility tests[a] of the hypermucoviscous *K. pneumoniae* isolates[b] with ultra-long viscous string (> 20 mm)

Strain	AMP	SAM	TZP	KZ	CTT	CAZ	TX	FEP	ATM	ETP	IPM	AMK	CN	TOB	CIP	LEV	F	SXT
RJA360	16/R	4/S	≤4/S	≤4/S	≤4/S	≤1/S	≤1/S	≤1/S	≤1/S	≤0.5/S	≤1/S	≤2/S	≤1/S	≤1/S	≤0.25/S	≤0.25/S	≤16/S	≤20/S
RJF67-2	≥32/R	4/S	≤4/S	≤4/S	≤4/S	≤1/S	≤1/S	≤1/S	≤1/S	≤0.5/S	≤1/S	≤2/S	≤1/S	≤1/S	≤0.25/S	≤0.25/S	64/I	≤20/S
RJA2570	16/R	4/S	≤4/S	≤4/S	≤4/S	≤1/S	≤1/S	≤1/S	≤1/S	≤0.5/S	≤1/S	≤2/S	≤1/S	≤1/S	≤0.25/S	≤0.25/S	32/S	≤20/S
RJF999	16/R	4/S	≤4/S	≤4/S	≤4/S	≤1/S	≤1/S	≤1/S	≤1/S	≤0.5/S	≤1/S	≤2/S	≤1/S	≤1/S	≤0.25/S	≤0.25/S	32/S	≤20/S
RJA166	≥32/R	≥32/R	≤4/S	≥64/R	16/S	≥64/R	8/R	≤1/S	≥64/R	≤0.5/S	≤1/S	≤2/S	≤1/S	≤1/S	0.5/S	1/S	64/I	≤20/S
RJF271	16/R	4/S	≤4/S	≤4/S	≤4/S	≤1/S	≤1/S	≤1/S	≤1/S	≤0.5/S	≤1/S	≤2/S	≤1/S	≤1/S	≤0.25/S	≤0.25/S	64/I	≤20/S
RJA277	≥32/R	≥32/R	≤4/S	≥64/R	≤4/S	≤1/S	32/R	2/S	2/S	≤0.5/S	≤1/S	4/S	≤1/S	≤1/S	≥4/R	≥8/R	≤16/S	≤20/S
RJA304	16/R	4/S	≤4/S	≤4/S	≤4/S	≤1/S	≤1/S	≤1/S	≤1/S	≤0.5/S	≤1/S	≤2/S	≤1/S	≤1/S	≤0.25/S	≤0.25/S	≤16/S	≤20/S
RJB442	16/R	4/S	≤4/S	≤4/S	≤4/S	≤1/S	≤1/S	≤1/S	≤1/S	≤0.5/S	≤1/S	≤2/S	≤1/S	≤1/S	≤0.25/S	≤0.25/S	≤16/S	≤20/S
RJF293	16/R	4/S	≤4/S	≤4/S	≤4/S	≤1/S	≤1/S	≤1/S	≤1/S	≤0.5/S	≤1/S	≤2/S	≤1/S	≤1/S	≤0.25/S	≤0.25/S	128R	≤20/S
RJF294	16/R	4/S	≤4/S	≤4/S	≤4/S	≤1/S	≤1/S	≤1/S	≤1/S	≤0.5/S	≤1/S	≤2/S	≤1/S	≤1/S	≤0.25/S	≤0.25/S	128/R	≤20/S
RJA898	16/R	4/S	≤4/S	≤4/S	≤4/S	≤1/S	≤1/S	≤1/S	≤1/S	≤0.5/S	≤1/S	≤2/S	≤1/S	≤1/S	≤0.25/S	≤0.25/S	64/I	≤20/S
RJA2225	16/R	4/S	≤4/S	≤4/S	≤4/S	≤1/S	≤1/S	≤1/S	≤1/S	≤0.5/S	≤1/S	≤2/S	≤1/S	≤1/S	≤0.25/S	≤0.25/S	≤16/S	≤20/S
RJA1385	16/R	4/S	≤4/S	≤4/S	≤4/S	≤1/S	≤1/S	≤1/S	≤1/S	≤0.5/S	≤1/S	≤2/S	≤1/S	≤1/S	≤0.25/S	≤0.25/S	32/S	≤20/S
RJA1253	16/R	4/S	≤4/S	≤4/S	≤4/S	≤1/S	≤1/S	≤1/S	≤1/S	≤0.5/S	≤1/S	≤2/S	≤1/S	≤1/S	≤0.25/S	≤0.25/S	32/S	≤20/S
RJA1657	16/R	4/S	≤4/S	≤4/S	≤4/S	≤1/S	≤1/S	≤1/S	≤1/S	≤0.5/S	≤1/S	≤2/S	≤1/S	≤1/S	≤0.25/S	≤0.25/S	64/I	≤20/S
RJA565	16/R	4/S	≤4/S	≤4/S	≤4/S	≤1/S	≤1/S	≤1/S	≤1/S	≤0.5/S	≤1/S	≤2/S	≤1/S	≤1/S	≤0.25/S	≤0.25/S	32/S	≤20/S
RJA1547	16/R	≤2/S	≤4/S	≤4/S	≤4/S	≤1/S	≤1/S	≤1/S	≤1/S	≤0.5/S	2/I	4/S	≤1/S	≤1/S	≤0.25/S	≤0.25/S	64/I	≤20/S
RJA1504	16/R	4/S	≤4/S	≤4/S	≤4/S	≤1/S	≤1/S	≤1/S	≤1/S	≤0.5/S	≤1/S	≤2/S	≤1/S	≤1/S	≤0.25/S	≤0.25/S	64/I	≤20/S
RJA1887	16/R	≤2/S	≤4/S	≤4/S	≤4/S	≤1/S	≤1/S	≤1/S	≤1/S	≤0.5/S	≤1/S	≤2/S	≤1/S	≤1/S	≤0.25/S	≤0.25/S	64/I	≤20/S
RJA726	16/R	4/S	≤4/S	≤4/S	≤4/S	≤1/S	≤1/S	≤1/S	≤1/S	≤0.5/S	≤1/S	≤2/S	≤1/S	≤1/S	≤0.25S	≤0.25/S	128/R	≤20/S

[a] The minimal inhibitory concentration was determined by the VITEK2 compact system. AMP, ampicillin; SAM, ampicillin/sulbactam; TZP, piperacillin/tazobactam; KZ, cefazolin; CTT, cefotetan; CAZ, ceftazidime; TX, ceftriaxone; FEP, cefepime; ATM, aztreonam;ETP, ertapenem; IPM, imipenem; AMK, amikacin; CN, gentamycin; TOB, tobramycin; CIP, ciprofloxacin; LEV, levofloxacin; F, ntrofurantoin; SXT, trimethoprim/sulfamethoxazole.

[b] These isolates include the 20 hypermucoviscous *Klebsiella pneumoniae* isolated between September 2014 and March 2016, and RJA726. *K. pneumoniae* RJA726 was isolated from the same patient as RJF293, in April 2014.

Table S6. Putative virulence factor genes detected in the genome of *hypervirulence K. pneumoniae* of RJF293 (ST374 and K2 serotype) [a]

Locus tag	Length (aa)	Identities (%)	*Ha*-value	Hit description
**Chromosome**				
RJF2_RS01505	752	72.2	0.695	*fepA*, ferrienterobactin outer membrane transporter
RJF2_RS04305	305	77.6	0.774	*lpxC*, UDP-3-O-R-3-hydroxymyristoyl, -N-acetylglucosamine deacetylase
RJF2_RS04615	140	68.1	0.657	*gspG*, general secretion pathway protein G
RJF2_RS04810	341	65.1	0.645	*lpxD*, UDP-3-O-3-hydroxymyristoyl, glucosamine N-acyltransferase
RJF2_RS04820	262	67.6	0.676	*lpxA*, UDP-N-acetylglucosamine acyltransferase
RJF2_RS04905	271	69	0.69	*ilpA*, immunogenic lipoprotein A
RJF2_RS05030	192	91	0.91	*gmhA,* phosphoheptose isomerase
RJF2_RS05325	195	95.9	0.959	yagZ/ecpA, *E. coli* common pilus structural subunit EcpA
RJF2_RS05330	222	91.4	0.914	*yagY*/*ecpB*, *E. coli* common pilus chaperone EcpB
RJF2_RS05335	841	93.3	0.933	*yagX*/*ecpC*, *E. coli* common pilus usher EcpC
RJF2_RS05340	547	94.1	0.941	*yagW*/*ecpD*, polymerized tip adhesin of ECP fibers
RJF2_RS05345	236	86.9	0.869	*yagV*/*ecpE*, *E. coli* common pilus chaperone EcpE
RJF2_RS06870	742	81.4	0.823	*fepA*, ferrienterobactin outer membrane transporter
RJF2_RS06875	402	66.4	0.659	*fes*, enterobactin/ferric enterobactin esterase
RJF2_RS06885	1293	78.1	0.78	*entF*, enterobactin synthase multienzyme complex component, ATP-dependent
RJF2_RS06890	264	88.6	0.883	*fepC*, ferrienterobactin ABC transporter ATPase
RJF2_RS06895	330	83	0.83	*fep*G, iron-enterobactin ABC transporter permease
RJF2_RS06900	335	86.2	0.857	*fep*D, ferrienterobactin ABC transporter permease
RJF2_RS06905	413	84.5	0.847	*entS*, enterobactin exporter, iron-regulated
RJF2_RS06910	319	84.3	0.79	*fepB*, ferrienterobactin ABC transporter periplasmic binding protein
RJF2_RS06915	391	75.4	0.754	*ent*C, isochorismate synthase 1
RJF2_RS06920	535	81.1	0.802	*entE*, 2,3-dihydroxybenzoate-AMP ligase component of enterobactin synthase multienzyme complex
RJF2_RS06925	283	87.3	0.876	*entB*, isochorismatase
RJF2_RS06930	251	88.7	0.876	*entA*, 2,3-dihydro-2,3-dihydroxybenzoate dehydrogenase

RJF2_RS08655	582	66.4	0.66	*msbA*, lipid transporter ATP-binding/permease
RJF2_RS08675	248	66.5	0.673	*kdsB*, 3-deoxy-manno-octulosonate cytidylyltransferase
RJF2_RS08865	356	85.4	0.854	*ompA*, outer membrane protein A
RJF2_RS09005	338	99.7	0.997	*kfuA*, iron ABC transporter substrate-binding protein
RJF2_RS09010	524	99.6	0.996	*kfuB*, iron ABC transporter permease
RJF2_RS09015	342	100	1	*kfuC*, Fe(3+) ions import ATP-binding protein
RJF2_RS09560	729	98.6	0.986	*iutA*, Ferric aerobactin receptor precusor
RJF2_RS13305	226	70.9	0.699	*mgtC*, Mg2+ transport protein
RJF2_RS14205	193	66.5	0.658	*sodB*, superoxide dismutase
RJF2_RS15420	300	74.4	0.717	*galU*, glucosephosphate uridylyltransferase
RJF2_RS15580	284	83	0.827	*kdsA*, 2-dehydro-3-deoxyphosphooctonate aldolase
RJF2_RS16675	434	98.4	0.984	*ybtS*, salicylate synthase Irp9
RJF2_RS16680	426	98.4	0.984	*ybtX*, putative signal transducer
RJF2_RS16685	600	99.8	0.998	*ybtQ*, inner membrane ABC-transporter YbtQ
RJF2_RS16690	570	99.6	0.996	*ybtP*, lipoprotein inner membrane ABC-transporter
RJF2_RS16695	319	99.4	0.994	*ybtA*, transcriptional regulator YbtA
RJF2_RS16700	2035	99.5	0.995	*irp2*, yersiniabactin biosynthetic protein
RJF2_RS16705	3163	99.5	0.995	*irp1*, yersiniabactin biosynthetic protein
RJF2_RS16710	366	99.5	0.995	*ybtU*, yersiniabactin biosynthetic protein YbtU
RJF2_RS16715	267	98.9	0.989	*ybtT*, yersiniabactin biosynthetic protein YbtT
RJF2_RS16720	525	99.4	0.994	*ybtE*, yersiniabactin siderophore biosynthetic protein
RJF2_RS17085	334	97.9	0.979	*uge*, uridine diphosphate galacturonate 4-epimerase (LPS gene cluster)
RJF2_RS17105	456	73.2	0.732	*manB*, phosphomannomutase
RJF2_RS19275	191	64.4	0.644	*algU*, alginate biosynthesis protein AlgZ/FimS
RJF2_RS19855	171	73.1	0.731	*luxS*, S-ribosylhomocysteinase [AI-2 (VF0406)]
RJF2_RS20220	343	65.2	0.644	*chuS*, heme oxygenase ChuS
RJF2_RS21105	234	100	1	*mrkH*, c-di-GMP-Dependent Transcriptional Activator

RJF2_RS21110	194	99.5	0.995	*mrkI*, transcriptional regulator
RJF2_RS21115	238	93.2	0.929	*mrkJ*, regulator of Type III fimbriae
RJF2_RS21120	211	100	1	*mrkF*, type 3 fimbriae anchor protein
RJF2_RS21125	331	100	1	*mrkD*, type 3 fimbriae adhesin
RJF2_RS21130	828	100	1	*mrkC*, type 3 fimbriae usher protein
RJF2_RS21135	233	100	1	*mrkB*, type 3 fimbriae assembly chaperone protein
RJF2_RS21140	202	99.5	0.995	*mrkA*, type 3 fimbriae major subunit
RJF2_RS21165	201	83.5	0.831	*fimB*, type 1 fimbriae regulatory protein fimB
RJF2_RS21170	202	82.8	0.787	*fimE*, type 1 fimbriae regulatory protein fimE
RJF2_RS21180	182	81.3	0.813	*fimA*, type-1 fimbrial protein, A chain precursor
RJF2_RS21335	207	100	1	*kvgA*, DNA-binding transcriptional activator
RJF2_RS21340	1214	99.6	0.996	*kvgS*, hybrid sensor histidine kinase
RJF2_RS22025	477	70.2	0.686	*rfaE*, ADP-heptose synthase
RJF2_RS24595	310	78.2	0.777	*rfaD*, ADP-L-glycero-D-mannoheptose-6-epimerase
**Plasmid**				
RJF2_RS26130	210	100	1	*rmpA*, regulator of mucoid phenotype
RJF2_RS26145	726	100	0.997	*iroN*, iron outer membrane receptor
RJF2_RS26150	409	100	1	*iroD*, salmochelin siderophore ferric enterochelin esterase
RJF2_RS26155	1214	99.9	0.999	*iroC*, salmochelin siderophore ATP-binding cassette
RJF2_RS26160	371	100	1	*iroB*, salmochelin siderophore glycosyltransferase
RJF2_RS26550	574	100	1	*iucA*, aerobactin siderophore biosynthesis protein
RJF2_RS26555	315	100	1	*iucB*, aerobactin siderophore biosynthesis protein
RJF2_RS26560	577	100	1	*iucC*, aerobactin siderophore biosynthesis protein
RJF2_RS26565	425	100	1	*iucD*, aerobactin siderophore biosynthesis protein
RJF2_RS26570	733	89.6	0.895	*iutA*, ferric aerobactin receptor precusor
RJF2_RS26605	114	94.2	0.851	*rmpA2*, regulator of mucoid phenotype

[a] Detection the putative virulence factors with the protein sequence similarities by using BLASTp-based H_a-value

To examine the degree of sequence similarities at an amino acid level between each query protein and the VRprofile-collected virulence factors, the NCBI BLASTp-derived H_a-value was employed.[5] For each query, the H_a-value was calculated as follows:

$$H_{\mathrm{a}} = i \times \frac{lm}{lq}$$

where i was the level of BLASTp identities of the region with the highest Bit score expressed as a frequency of between 0 and 1, l_m the length of the highest scoring matching sequence (including gaps) and l_q the query length. If there were no matching sequences with a BLASTp E value < 0.01, the H_a-value assigned to that query sequence was defined as zero. Therefore H_a-value belonged to the set, $H_a \in [0,1]$. Here, a strict H_a-value cut-off ≥ 0.64 was used to determine the significant sequence similarities; for example, the identities is 80% and the ratio of matching length is 80%.

Table S7. List of the putative virulence factors detected in the 106 *K. pneumoniae* complete genome sequences

#	Gene	Product
1	*allA*	Ureidoglycolate hydrolase
2	*allB*	Allantoinase
3	*allC*	Allantoate amidohydrolase
4	*allD*	Ureidoglycolate dehydrogenase
5	*allR*	Regulator of the allantoin metabolism gene cluster
6	*allS*	Transcriptional activator of the allantoin metabolism gene cluster
7	*arcC*	Carbamate kinase
8	*fdrA*	NAD(P)-binding acyl-CoA synthetase
9	*gcl*	Glyoxylate carboligase
10	*glxK*	Glycerate kinase
11	*glxR*	Tartronic semialdehyde reductase
12	*hyi*	Hydroxypyruvate isomerase
13	*KP1_1364*	Probable metabolite transport protein
14	*KP1_1371*	Putative glyoxylate utilization gene
15	*ybbW*	Allantoin permease
16	*ybbY*	Purine permease
17	*ylbE*	Putative cytoplasmic protein
18	*ylbF*	Anaerobic allantoin catabolic oxamate carbamoyltransferase
19	*rmpA*	Regulator of mucoid phenotype
20	*rmpA2*	Regulator of mucoid phenotype
21	*kvgA*	DNA-binding transcriptional activator
22	*kvgS*	Hybrid sensor histidine kinase
23	*iroB*	Salmochelin siderophore glycosyltransferase
24	*iroC*	Salmochelin siderophore ATP-binding cassette
25	*iroD*	Salmochelin siderophore ferric enterochelin esterase
26	*iroN*	Iron outer membrane receptor
27	*irp1*	Yersiniabactin biosynthetic protein
28	*irp2*	Yersiniabactin biosynthetic protein
29	*iucA*	Aerobactin siderophore biosynthesis protein
30	*iucB*	Aerobactin siderophore biosynthesis protein
31	*iucC*	Aerobactin siderophore biosynthesis protein
32	*iucD*	Aerobactin siderophore biosynthesis protein
33	*iutA14*	Ferric aerobactin receptor precusor
34	*kfuA*	Iron ABC transporter substrate-binding protein
35	*kfuB*	Iron ABC transporter permease
36	*kfuC*	Fe(3+) ions import ATP-binding protein
37	*mrkA*	Type 3 fimbriae major subunit
38	*mrkB*	Type 3 fimbriae assembly chaperone protein
39	*mrkC*	Type 3 fimbriae usher protein

40	*mrkD*	Type 3 fimbriae adhesin
41	*mrkF*	Type 3 fimbriae anchor protein
42	*mrkH*	c-Di-GMP-Dependent Transcriptional Activator
43	*mrkI*	Transcriptional regulator
44	*mrkJ*	Regulator of Type III fimbriae
46	*algU*	Alginate biosynthesis protein AlgZ/FimS
47	*rfaE*	ADP-heptose synthase
48	*rfaD*	ADP-L-glycero-D-mannoheptose-6-epimerase
49	*ybtE*	Yersiniabactin siderophore biosynthetic protein
50	*ybtT*	Yersiniabactin biosynthetic protein YbtT
51	*ybtU*	Yersiniabactin biosynthetic protein YbtU
52	*ybtA*	Transcriptional regulator YbtA
53	*ybtP*	lipoprotein inner membrane ABC-transporter
54	*ybtQ*	Inner membrane ABC-transporter YbtQ
55	*ybtX*	Putative signal transducer
56	*ybtS*	Salicylate synthase Irp9
57	*mgtB*	Mg2+ transport protein
58	*mgtC*	Mg2+ transport protein
59	*fimB*	Type 1 fimbriae Regulatory protein fimB
60	*fimE*	Type 1 fimbriae Regulatory protein fimE
61	*fimA*	Type-1 fimbrial protein, A chain precursor
62	*fimI*	Fimbrin-like protein fimI precursor
63	*fimC*	Chaperone protein fimC precursor
64	*fimD*	Outer membrane usher protein fimD precursor
65	*fimF*	FimF protein precursor
66	*fimG*	FimG protein precursor
67	*fimH*	FimH protein precursor
68	*chuS*	Heme oxygenase ChuS
69	*fepA*	Ferrienterobactin outer membrane transporter
70	*fepB*	Ferrienterobactin ABC transporter periplasmic binding protein
71	*fepC*	Ferrienterobactin ABC transporter ATPase
72	*fepD*	Ferrienterobactin ABC transporter permease
73	*fepG*	Iron-enterobactin ABC transporter permease
74	*entF*	Enterobactin synthase multienzyme complex component, ATP-dependent
75	*entC*	Isochorismate synthase 1
76	*entE*	2,3-Dihydroxybenzoate-AMP ligase component of enterobactin synthase multienzyme complex
77	*entB*	isochorismatase
78	*entA*	2,3-Dihydro-2,3-dihydroxybenzoate dehydrogenase
79	*ompA*	Outer membrane protein A
80	*sodB*	Superoxide dismutase
81	*gspG*	General secretion pathway protein G
82	*manB*	Phosphomannomutase

83	*fcl*	GDP-fucose synthetase [O-antigen (VF0392)]
84	*gmd*	GDP-mannose 4,6-dehydratase [O-antigen (VF0392)]
85	*yagY*	*E. coli* common pilus chaperone EcpB
86	*gmhA*	Phosphoheptose isomerase
87	*yagZ*	*E. coli* common pilus structural subunit EcpA
88	*yagX*	*E. coli* common pilus usher EcpC
89	*yagW*	Polymerized tip adhesin of ECP fibers
90	*yagV*	*E. coli* common pilus chaperone EcpE
91	*kdsB*	3-Deoxy-manno-octulosonate cytidylyltransferase
92	*msbA*	Lipid transporter ATP-binding/permease
93	*galU*	Glucosephosphate uridylyltransferase
94	*lpxD*	UDP-3-O-(3-hydroxymyristoyl) glucosamine N-acyltransferase; Lipopolysaccharides
95	*lpxA*	UDP-N-acetylglucosamine acyltransferase; Lipopolysaccharides
96	*lpxC*	UDP-3-O-(R-3-hydroxymyristoyl)-N-acetylglucosamine deacetylase; Lipopolysaccharides
97	*kdsA*	2-Dehydro-3-deoxyphosphooctonate aldolase
98	*luxS*	S-ribosylhomocysteinase
99	*fes*	Enterobactin/ferric enterobactin esterase
100	*entS*	Enterobactin exporter, iron-regulated
101	*IlpA*	Immunogenic lipoprotein A
102	*uge*	Uridine diphosphate galacturonate 4-epimerase (LPS synthesis gene cluster)
103	*clbB*	Putative peptide/polyketide synthase (colibactin synthesis gene cluster clbB-Q)
104	*clbN*	Putative non-ribosomal peptide synthetase (colibactin synthesis gene cluster clbB-Q)
105	*clbQ*	Iron aquisition yersiniabactin synthesis enzyme (colibactin synthesis gene cluster clbB-Q)
106	*mceC*	Microcin E492 modification (microcin E492 synthesis gene cluster)
107	*mceD*	Apo and ferric salmochelin esterase (microcin E492 synthesis gene cluster)
108	*mceJ*	mMicrocin E492 modification with salmochelin (microcin E492 synthesis gene cluster)
109	*mceI*	Microcin E492 modification with salmochelin (microcin E492 synthesis gene cluster)
110	*mceH*	Second component of microcin E492 exporter (microcin E492 synthesis gene cluster)
111	*mceG*	ABC protein of microcin E492 exporter (microcin E492 synthesis gene cluster)
112	*mceF*	Similar to McmM of CAAX amino terminal protease family (microcin E492 synthesis gene cluster)

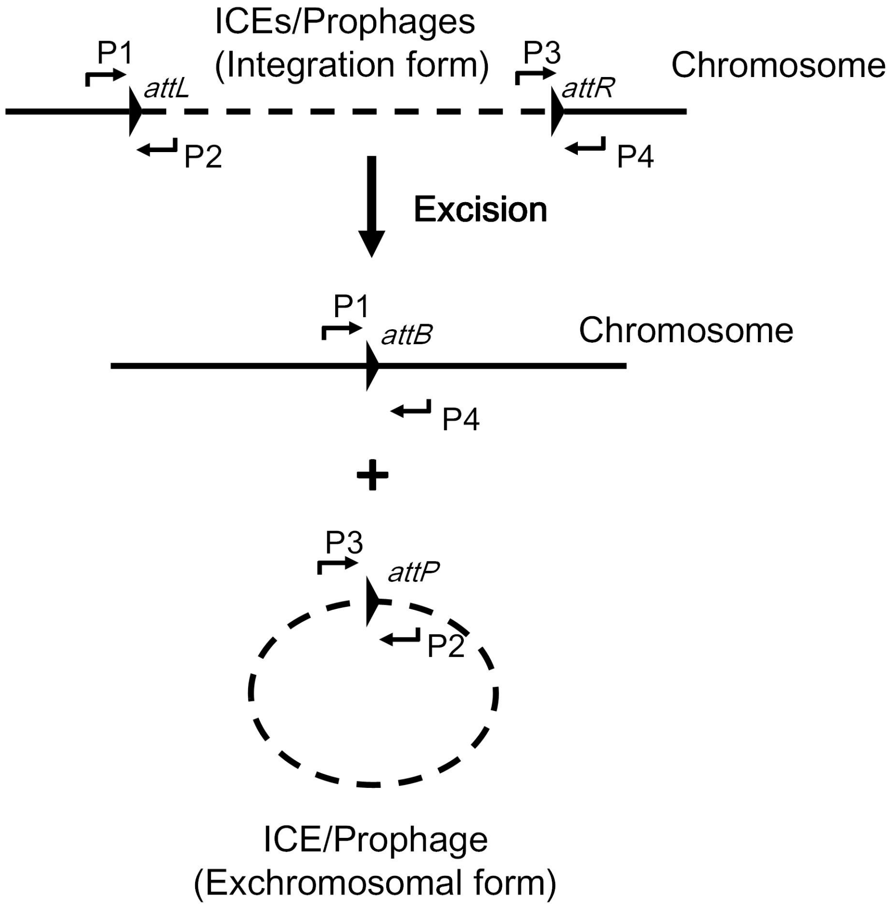

Figure S1. Detection of the ICE or prophage excision by PCR. The excision of ICE or prophage can result in a *attB* site on the chromosome. PCR assay with primers outside the *attL* and *attR* sites (P1 and P4 in the diagram) can be employed to amplify the *attB* site upon the ICE or prophage excision. No amplicon can be obtained with this pair of primers if the ICE or prophage remains integrated in the chromosome, as the size of ICE or prophage is outside the range of DNA polymerase capability.

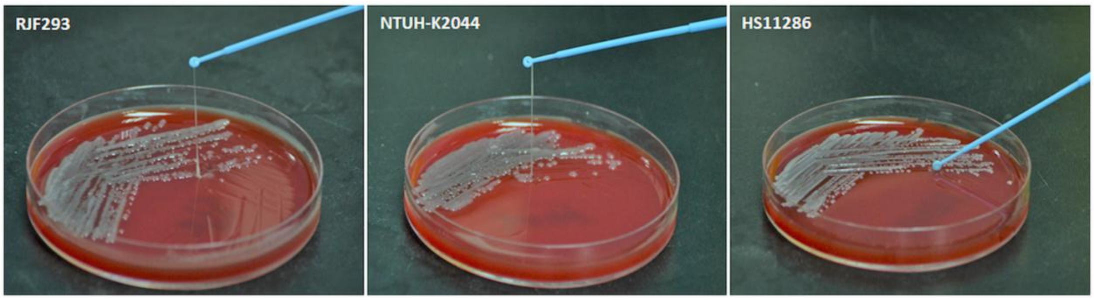

Figure S2. Positive hypermucoviscosity phenotype of *K. pneumoniae* RJF293 by string test. The K1 and ST23 hvKP strain NTUH-K2044 was used for positive control while the ST11 cKP strain HS11286 for negative control.

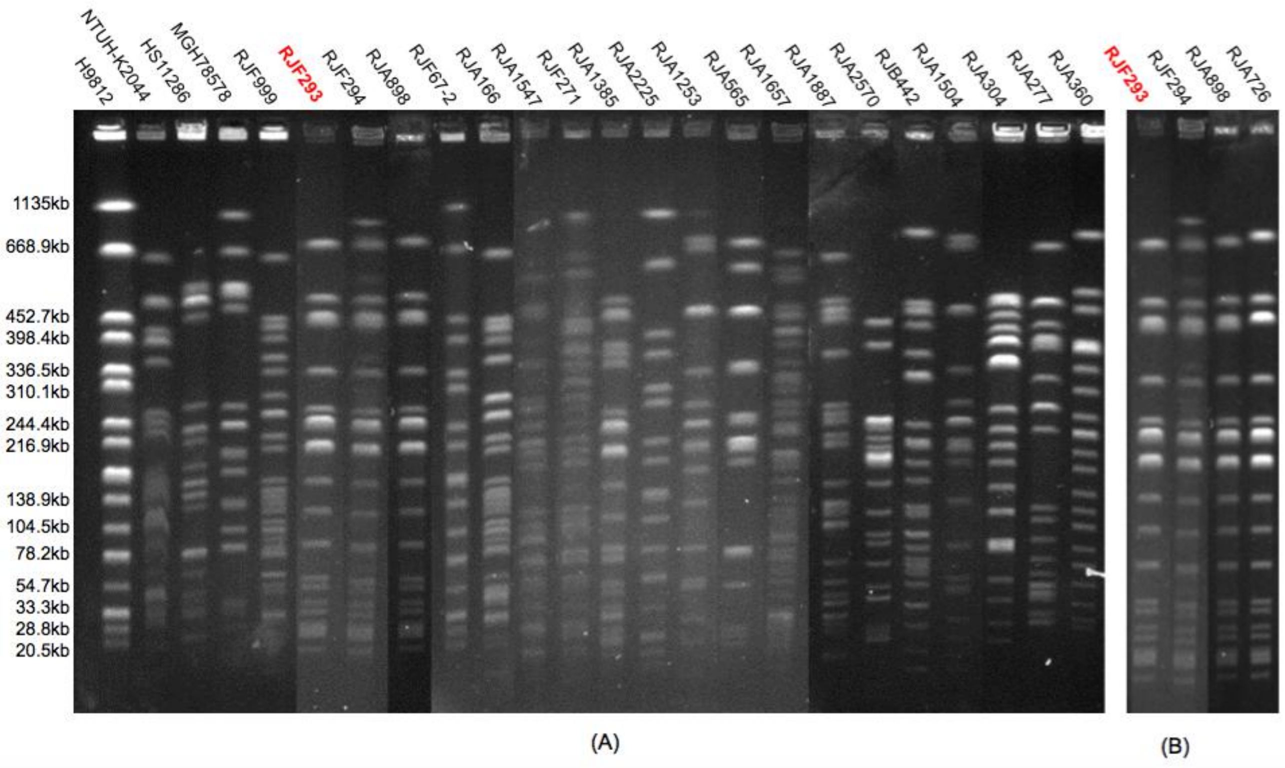

Figure S3. Gel image of PFGE for hypermucoviscous *K. pneumoniae* clinical isolates. (A) PFGE analysis of the 20 hypermucoviscous *K. pneumoniae* isolates collected from September 2014 to March 2016. Four reference isolates, *Salmonella* serotype Braenderup H9812, *K. pneumoniae* NTUH-K2044, HS11286 and MGH78578*,* were used as controls. Genomic DNA was digested with *Xba*I and subjected to pulsed-field gel electrophoresis (PFGE). (B)The four isolates (RJF293, RJF294, RJA898 and RJA726) collected from the same patient showed same PFGE patterns.

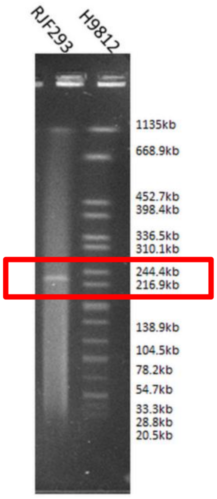

Figure S4. Gel image of S1-PFGE for *K. pneumoniae* RJF293. Genomic DNA was digested using S1-nuclease and subjected to pulsed-field gel electrophoresis. The band marked in a red rectangle is corresponding to the plasmid pRJF293.

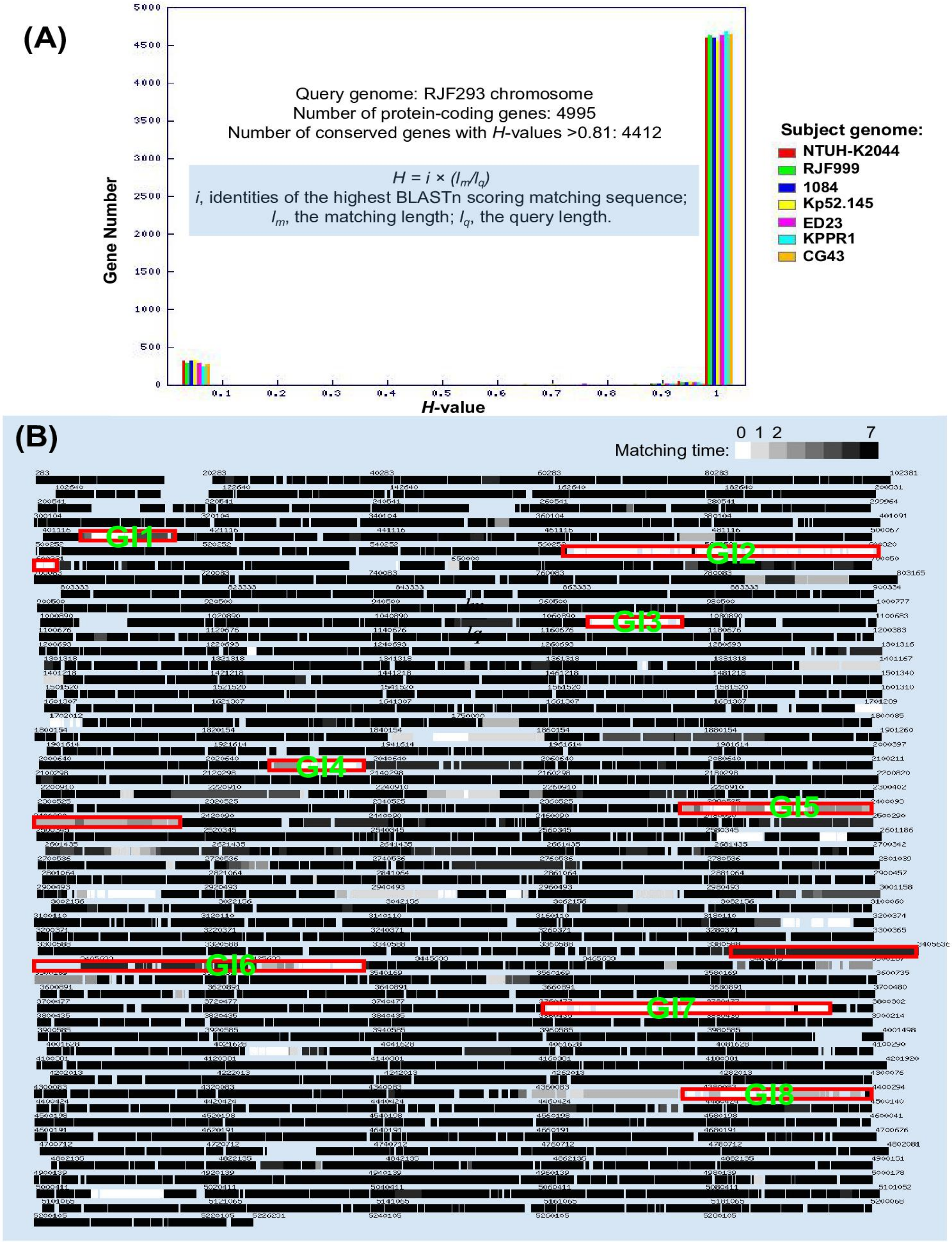

Figure S5. The *in silico* subtractive hybridization of the *K. pneumoniae* RJF293 genome against genomes of other seven completely sequenced hvKP isolates by using mGenomeSubtractor.[7] The seven subject hvKP chromosomes include (supplementary Table S4): 1084, CG43, ED23, Kp52.145, KPPR1, NTUH-K2044 and RJF999. **(A)** Histogram of BLASTn-based *H*-values for all RJF293 genes against all seven subject chromosome sequences (color-coded). **(B)** Chromosome map of RJF293 with gene black/white-shade-coded based on the number of comparator *K. pneumoniae* genomes identified as harboring a nucleotide sequence-conserved homolog. Genes shown in absolute black ('7') are conserved across all seven *K. pneumoniae* comparator genomes, with genes shown in decreasing shades of black being conserved in lower numbers of comparator genomes, while at the other extreme those shown in white ('0') are unique to RJF293. Non-coding regions are shown as gaps. The genomic island-like variable regions (also see Table 2) are marked by red rectangles

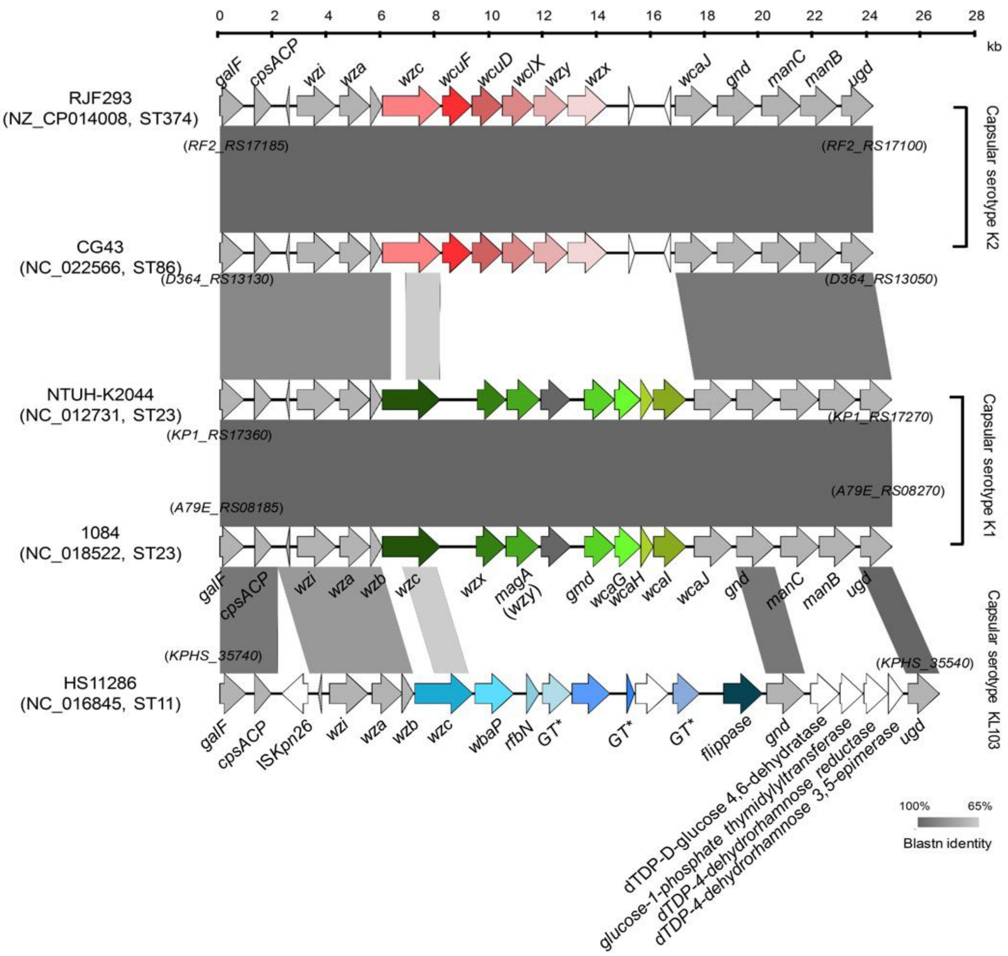

Figure S6. Alignment maps of the five representative *K. pneumoniae* CPS gene clusters. The *cps* gene cluster was located between *galF* and *ugd* as reported by the previous study. The conserved protein-coding genes are presented in grey. Variable regions are differentiated by colors. GT, glycosyl transferas

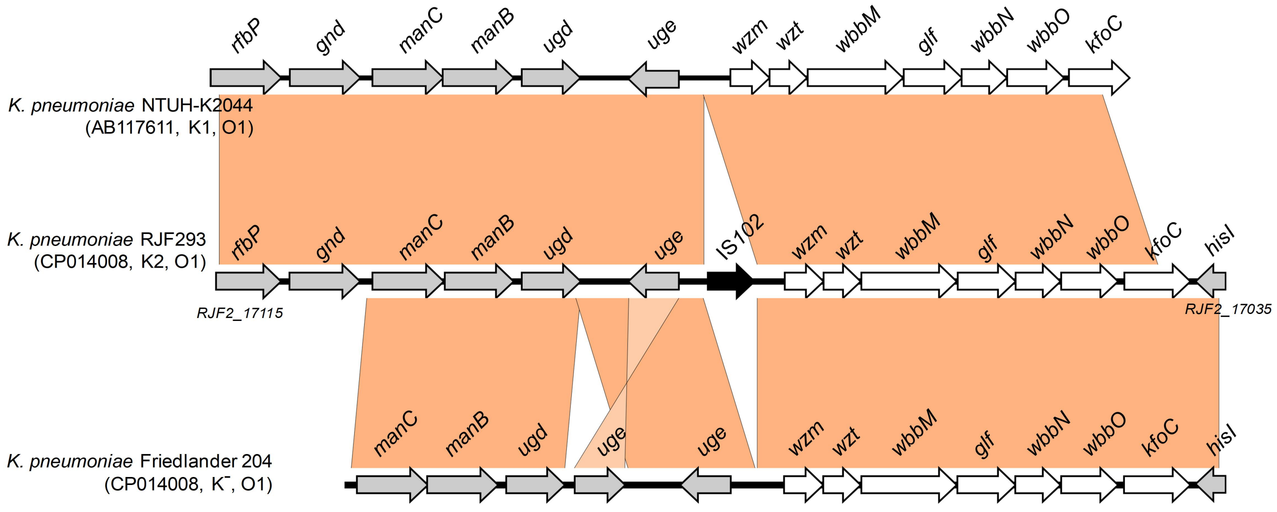

Figure S7. Alignments between the O1 LPS gene cluster in *K. pneumoniae* NTUH-K2044, RJF293 and Friedlander 204.

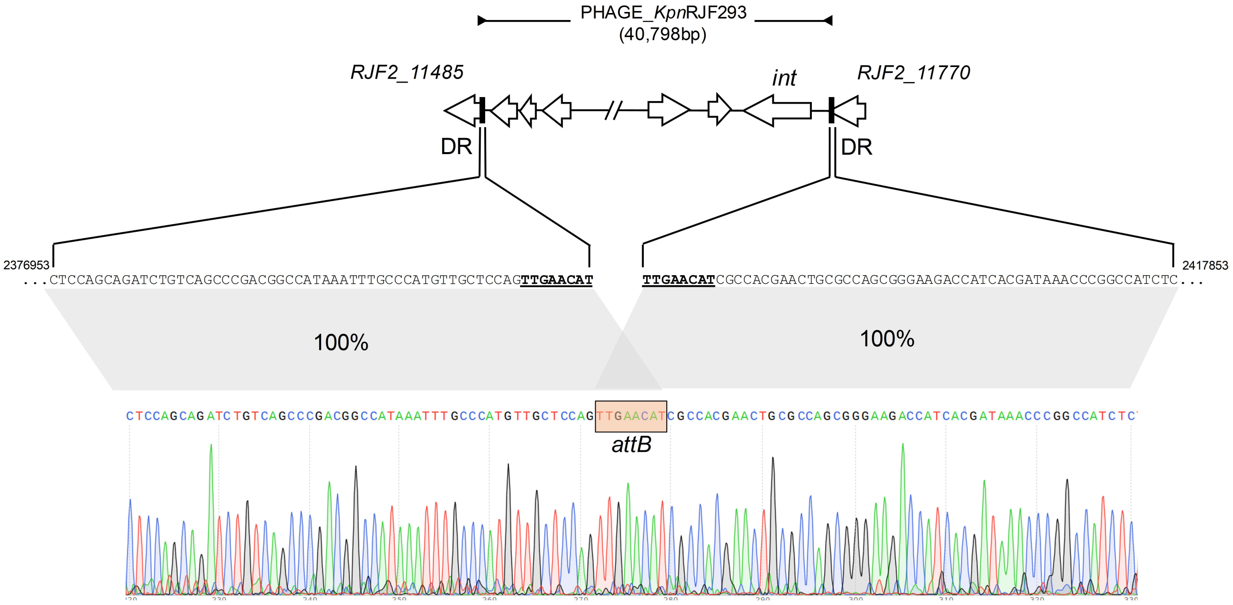

Figure S8 Excision of PHAGE_*Kpn*RJF293 from the RJF293 chromosome. The *attB* site sequence was determined to be TTGAACAT. The chromatogram shows the sequencing result of *attB* site and its flanking region after prophage excision. The exact *attB* site determined by DNA sequencing was highlighted with yellow background. The sites *attL* and *attR* (named according to the integrase gene orientation) were shown in bold and underlined. The sequences flanking *attB* were 100% identical to the sequences outside *attR* and *attL* sites, indicated by the grey parallelogram.

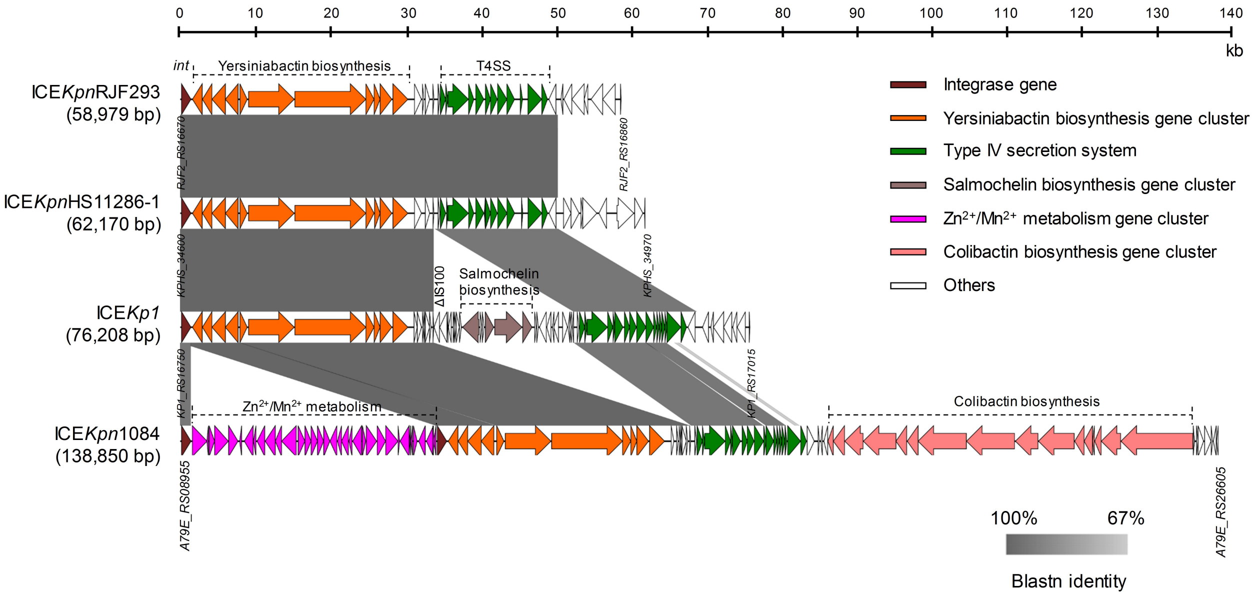

Figure S9. Sequence alignments between four ICE*Kp*1 family ICEs. ICE*Kpn*RJF293, ICE*Kp1,* and ICE*Kpn*1084 are located respectively on the chromosomes of the hvKP strain RJF293, NTUH-K2044, and 1084. ICE*Kpn*HS11286-1 is carried by the chromosome of the cKP strain HS11286. All of the four ICEs harbored the integrase gene, yersiniabactin biosynthesis gene cluster, and Type-F type IV secretion system gene cluster. The regions in the 3'-end of ICEs are variable among these strains.

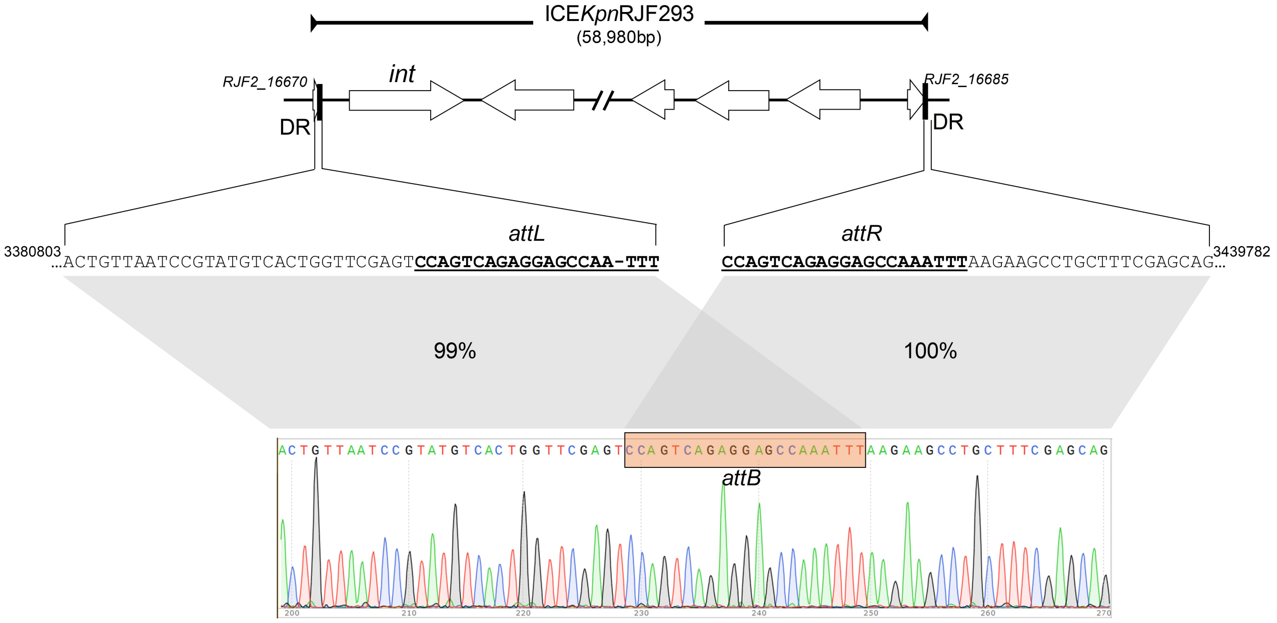

Figure S10. Site-specifically excision of ICE*Kpn*RJF293 from the 3'-end of the tRNA^{Asn} gene on the RJF293 chromosome. The *attB* site sequence was determined to be CCAGTCAGAGGAGCCAAATTT. The chromatogram shows the sequencing result of *attB* site and its flanking region after ICE excision. The exact *attB* site determined by DNA sequencing was highlighted with yellow background. The sites *attL* and *attR* (named according to the integrase gene orientation) were shown in bold and underlined. The sequences flanking *attB* were 100% identical to the sequences outside *attL* and *attR* sites, indicated by the grey parallelogram.

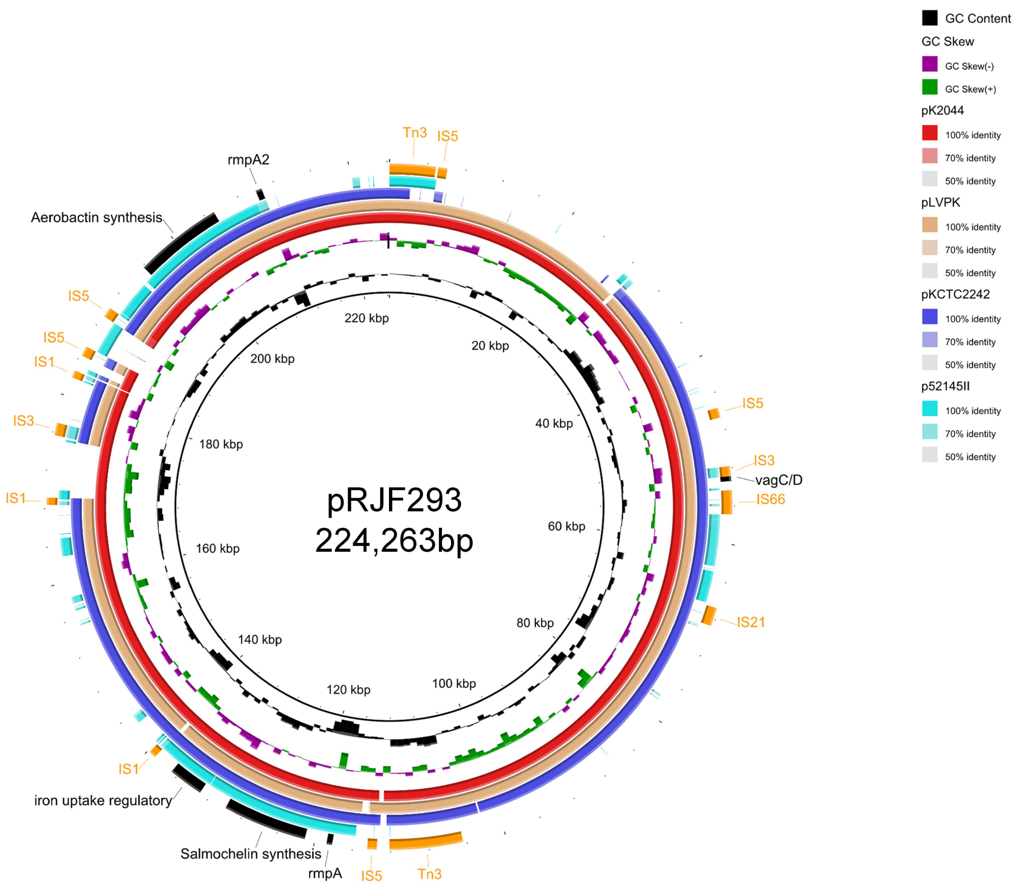

Figure S11. Diagram of the plasmid pRJF293 and the sequence alignments to four other completely sequenced *K. pneumoniae* virulence plasmids: pK2044 (224,152 bp in size), pLVPK (219,385 bp), pKCTC2242 (202,852 bp) and p52.145II (121,703 bp).

REFERENCES

1. Wu KM, Li LH, Yan JJ, Tsao N, Liao TL, Tsai HC, Fung CP, Chen HJ, Liu YM, Wang JT, et al. Genome sequencing and comparative analysis of *Klebsiella pneumoniae* NTUH-K2044, a strain causing liver abscess and meningitis. J Bacteriol 2009; 191:4492–501.
2. Wyres KL, Wick RR, Gorrie C, Jenney A, Follador R, Thomson NR, Holt KE. Identification of *Klebsiella* capsule synthesis loci from whole genome data. Microb Genom 2016; 2:e000102.
3. Jolley KA, Maiden MC. BIGSdb: Scalable analysis of bacterial genome variation at the population level. BMC Bioinformatics 2010; 11:595.
4. Shon AS, Bajwa RPS, Russo TA. Hypervirulent (hypermucoviscous) *Klebsiella pneumoniae*: a new and dangerous breed. Virulence 2013; 4:107–18.
5. Li J, Tai C, Deng Z, Zhong W, He Y, Ou H-Y. VRprofile: gene-cluster-detection-based profiling of virulence and antibiotic resistance traits encoded within genome sequences of pathogenic bacteria. Brief Bioinform 2017; bbw141
6. Edgar RC. PILER-CR: fast and accurate identification of CRISPR repeats. BMC Bioinformatics 2007; 8:18.
7. Shao Y, He X, Harrison EM, Tai C, Ou H-Y, Rajakumar K, Deng Z. mGenomeSubtractor: a web-based tool for parallel in silico subtractive hybridization analysis of multiple bacterial genomes. Nucleic Acids Res 2010; 38:W194-200.
